# Identification of Hypoxia‐*ALCAM* ^high^ Macrophage‐ Exhausted T Cell Axis in Tumor Microenvironment Remodeling for Immunotherapy Resistance

**DOI:** 10.1002/advs.202309885

**Published:** 2024-07-02

**Authors:** Zhenzhen Xun, Huanran Zhou, Mingyi Shen, Yao Liu, Chengcao Sun, Yanhua Du, Zhou Jiang, Liuqing Yang, Qing Zhang, Chunru Lin, Qingsong Hu, Youqiong Ye, Leng Han

**Affiliations:** ^1^ Center for Immune‐Related Diseases at Shanghai Institute of Immunology Department of Gastroenterology Ruijin Hospital Shanghai Jiao Tong University School of Medicine Shanghai 200025 China; ^2^ Shanghai Institute of Immunology State Key Laboratory of Systems Medicine for Cancer Department of Immunology and Microbiology Shanghai Jiao Tong University School of Medicine Shanghai 200025 China; ^3^ Department of Endocrinology The First Affiliated Hospital of USTC Division of Life Sciences and Medicine University of Science and Technology of China Hefei Anhui 230001 China; ^4^ Department of Hepatobiliary Surgery Centre for Leading Medicine and Advanced Technologies of IHM The First Affiliated Hospital of USTC Division of Life Sciences and Medicine University of Science and Technology of China Hefei 230001 China; ^5^ Department of Molecular and Cellular Oncology The University of Texas MD Anderson Cancer Center Houston TX 77030 USA; ^6^ Simmons Comprehensive Cancer Center Department of Pathology University of Texas Southwestern Medical Center Dallas TX 75390 USA; ^7^ Brown Center for Immunotherapy School of Medicine Indiana University Indianapolis IN 46202 USA; ^8^ Department of Biostatistics and Health Data Science School of Medicine Indiana University Indianapolis IN 46202 USA; ^9^ Department of Biochemistry and Molecular Biology McGovern Medical School at The University of Texas Health Science Center at Houston Houston TX 77030 USA

**Keywords:** *ALCAM* ^+^ macrophage, cancer immunotherapy, exhausted T cell, hypoxia, spatial transcriptomics

## Abstract

Although hypoxia is known to be associated with immune resistance, the adaptability to hypoxia by different cell populations in the tumor microenvironment and the underlying mechanisms remain elusive. This knowledge gap has hindered the development of therapeutic strategies to overcome tumor immune resistance induced by hypoxia. Here, bulk, single‐cell, and spatial transcriptomics are integrated to characterize hypoxia associated with immune escape during carcinogenesis and reveal a hypoxia‐based intercellular communication hub consisting of malignant cells, *ALCAM* ^high^ macrophages, and exhausted CD8^+^ T cells around the tumor boundary. A hypoxic microenvironment promotes binding of HIF‐1α complex is demonstrated to the *ALCAM* promoter therefore increasing its expression in macrophages, and the *ALCAM* ^high^ macrophages co‐localize with exhausted CD8^+^ T cells in the tumor spatial microenvironment and promote T cell exhaustion. Preclinically, HIF‐1ɑ inhibition reduces *ALCAM* expression in macrophages and exhausted CD8^+^ T cells and potentiates T cell antitumor function to enhance immunotherapy efficacy. This study reveals the systematic landscape of hypoxia at single‐cell resolution and spatial architecture and highlights the effect of hypoxia on immunotherapy resistance through the *ALCAM* ^high^ macrophage‐exhausted T cell axis, providing a novel immunotherapeutic strategy to overcome hypoxia‐induced resistance in cancers.

## Graphical abstract

This work integrates bulk, single‐cell, and spatial transcriptomics to characterize hypoxia associated with immune escape during carcinogenesis. The results reveal the systematic landscape of hypoxia at single‐cell resolution and spatial architecture, and highlights the effect of hypoxia on immunotherapy resistance through the ALCAMhighmacrophage‐exhausted T cell axis, providing a novel immunotherapeutic strategy to overcome hypoxia‐induced resistance in cancers.

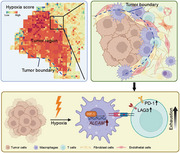

## 1 Introduction

The immune system plays a central role in the surveillance and elimination of malignant cells^[^ ^1^ ^]^ Nascent malignant cells escape immunosurveillance through diverse mechanisms, including reduced antigenicity that decreases recognition by the immune system, upregulation of immunoinhibitory molecules, such as PD‐L1, CTLA‐4, and TIM‐3,^[^ ^2^, ^3^, ^4^ ^]^ recruitment of immunosuppressive cells, such as tumor‐associated macrophages (TAMs), myeloid‐derived suppressor cells (MDSCs), and regulatory T cells (Tregs).^[^ ^5^, ^6^, ^7^, ^8^ ^]^ Multiple immune checkpoints (e.g., PD‐1, CTLA‐4) can inhibit functional T cell activation^[^ ^9^ ^]^ Current developments in immune checkpoint blockade (ICB) have shown promising clinical effects and suggested a number of antibodies targeting inhibitory checkpoints that might exhibit anti‐tumor immunity.^[^ ^10^, ^11^, ^12^ ^]^ However, the overall response rate is generally less than 40%.^[^ ^13^, ^14^, ^15^ ^]^ Selection of proper immune checkpoint inhibitors that are most suitable for certain cancer types remains elusive. Therefore, it is necessary to explore characteristics of the tumor microenvironment (TME) of patients and develop stratified immunotherapies to achieve enhanced therapeutic effects.

Hypoxia is a state of insufficient oxygen availability that occurs in solid tumors and is one of the most important TME features^[^ ^16^ ^]^ Rapid proliferation of tumor cells exerts stress on the oxygen supply, leading to hypoxic regions within the TME^[^ ^17^ ^]^ Cells can adapt and survive in the hypoxic state through intercellular regulation, including altered protein synthesis, energy metabolism, mitochondrial respiration, and nutrient utilization^[^ ^18^ ^]^ A recent study demonstrated that hypoxia‐inducible factor (HIF) can alter the ratio of CD4^+^ T cell subsets and their cytokine secretion^[^ ^19^ ^]^ CD8^+^ T cells in different stages show various level of hypoxia^[^ ^20^ ^]^ Moreover, TAMs massively infiltrate the hypoxic region, showing vessel formation function and immunosuppressive phenotype^[^ ^21^ ^]^ These studies demonstrate that immune cells are affected by hypoxia. However, most studies on the effects of hypoxia on the TME are focused on tumor cells or only a few cell types, thus neglecting the effects on various cell types and the complex crosstalk among cells in the TME. Single‐cell sequencing (scRNA‐seq) and spatial transcriptomics (ST) provide a unique opportunity to characterize the functional roles of hypoxia in the TME at single‐cell resolution and spatial architecture^[^ ^22^ ^]^ thus allowing for a better understanding of the heterogeneity of each cell type in a hypoxic TME and the hypoxia‐based crosstalk of different cell types.

In this study, we integrated bulk, spatial, and single‐cell transcriptomics to characterize the dynamic alterations of hypoxia status and the TME during tumorigenesis or under immunotherapy. We revealed that hypoxia is associated with the activated leukocyte cell adhesion molecule (*ALCAM*)^high^ macrophages enrichment and CD8^+^ T cell exhaustion around the tumor boundary in the tumor spatial microenvironment. Hypoxia can induce Hif1a binding at the *ALCAM* promoter to increase *ALCAM* expression in macrophages and is associated with exhausted CD8^+^/CD4^+^ T cell differentiation. Preclinically, we demonstrated that EZN‐2968, an antisense oligonucleotide inhibitor of hypoxia‐inducible factor‐1 alpha (HIF‐1α)^[^ ^23^ ^]^ can reduce *ALCAM* ^high^ macrophages and restore exhausted *CD8* ^+^ T cells, therefore demonstrating synergistic effects when combined with PD‐1 mAb in the treatment of melanoma and triple‐negative breast cancer (TNBC) immune competent mouse models. Our study highlights hypoxia‐associated immunotherapy resistance through the *ALCAM* ^high^ macrophage‐exhausted T cells (Tex) axis and provides a novel therapeutic strategy in immunotherapy.

## 2 Results

### 2.1 Characterization of the Dynamic Alterations of Tumor Hypoxia and Immune Microenvironment in Tumorigenesis and Immunotherapy Resistance

**Figure 1:**
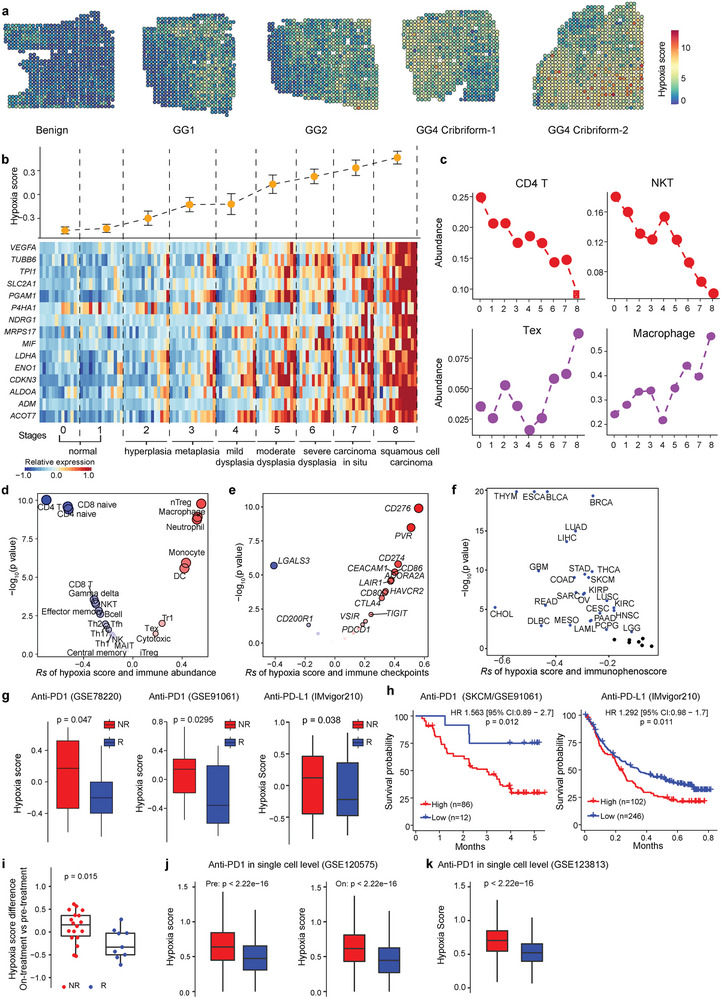
Dynamics of hypoxia and immune microenvironment during tumorigenesis and immunotherapy. a) Spatial feature plots show hypoxia status at different stages of prostate cancer in spatial transcription data (n = 10,5 × 5 mm). The samples with *Benign* annotation were totally histologically graded as benign, while samples annotated as *GG1*, *GG2*, *GG4 Cribriform* were consistent with malignant cells and partial benign cells. b) Continuous shift in hypoxia score (upper panel) during lung carcinogenesis. Results are displayed as mean ± s.d. Heat maps (bottom panel) show the expression level of 15 genes of hypoxia signature for each stage. c) The estimation of immune‐cell abundance of CD4 T, NKT, Tex, and macrophages, shows the evolving immune contexture for each developmental stage. Abbreviations: GG: Grade Group; NKT, natural killer T cells; nTreg, natural regulatory T cells; Tr1: type 1 regulatory cells; Tex: exhausted T cells. d) Spearman's correlation of hypoxia score with the abundance of 24 immune cell types (including 18 T‐cell subsets, 6 other important immune cells) from transcriptome data. Black circle indicates p < 0.05. e) Spearman's correlation between hypoxia score and the mRNA expression of 20 inhibitory immune checkpoints. Black circle indicates p < 0.05. f) Spearman's correlation of hypoxia score and immunophenoscore. Blue dots indicate p < 0.05. g) Hypoxia score difference between responders and non‐responders with treatment of PD‐1 or PD‐L1 inhibitors in three independent datasets, GSE78220, GSE91061, and IMvigor210 cohorts. h) Kaplan–Meier curves showing patients with higher hypoxia score are associated with worse overall survival in GSE91061 and IMvigor210 cohorts. n; number of patients. i) Alteration in hypoxia score between on‐treatment and pre‐treatment samples in responders and non‐responders. j,k) Hypoxia score difference between all cells of responders and non‐responders with PD‐1 inhibitors both in pre‐treatment and on‐treatment in single‐cell transcriptomics, including GSE120575 and GSE123813. A two‐sided log‐rank test was used to assess statistical significance in (h). An unpaired two‐sided Wilcoxon signed‐rank test was performed in (g, and i–k).

To investigate the functional roles of hypoxia in microenvironment remodeling in tumorigenesis, we collected spatial transcriptomics data of a patient's prostate tumor mid‐gland axial section covering the tumor from initiation to progression (Table S1, Supporting Information). The section was divided into several cubes and histologically graded following the Gleason grading system including benign, and the Gleason score ranges from Grade Group (GG)1 to GG5 (a higher score means that the cube looks more likely to be abnormal tissue). We calculated the hypoxia score as in our previous study^[^ ^25^ ^]^ in each spot (see Methods), and we found a higher hypoxia score in more aggressive tissues (**Figure** **1**a). Further, we collected two datasets of mRNA expression with continuous morphological stages of the carcinogenesis of lung squamous cell carcinoma (LSCC; GSE33479)^[^ ^26^ ^]^ and prostate cancer (GSE6099)^[^ ^27^ ^]^ (Table S2, Supporting Information). For LSCC, there are 122 carefully annotated biopsies from 77 patients with nine morphological stages, ranging from stages 0 and 1 that represent bronchial mucosa with normal histology, which had normal and low fluorescence, respectively, to stage 8 that represents segregated invasive tissue from premalignant lesions (see Methods for details). We observed that the hypoxia score gradually increases in carcinogenesis of LSCC, from stage 0 to stage 8 (Figure 1b). In addition, we observed a similar gradually increased hypoxic pattern in prostate cancer progression, ranging from stromal nodules of benign prostatic hyperplasia (STROMA), prostatic intraepithelial neoplasia (PIN), to prostate carcinoma (PCA), and metastatic prostate cancer (MET) (Figure S1a, Supporting Information). These results demonstrated increasing hypoxia status in tumorigenesis.

Hypoxia has been suggested to be involved in immune resistance^[^ ^28^ ^]^ but the crosstalk between hypoxia and the TME during tumorigenesis and the potential underlying molecular mechanisms remain elusive. We found that total tumor infiltration immune cells (Infiltration Score, see Methods) increased during LSCC carcinogenesis (Figure S1b, Supporting Information), and the infiltration score was correlated with the hypoxia score (*Rs* = 0.4; p = 4.8 × 10^−6^. Figure S1c, Supporting Information). To further characterize the immune response and immune escape from the immune infiltration and to understand the interactions between hypoxia status and various immune cell populations, we estimated the abundance of 24 immune cell types including 18 T‐cell subsets and B cells, macrophages, monocytes, neutrophils, dendritic cells (DC), and natural killer (NK) cells. We found that the hypoxia score negatively correlated with the abundance of 11 immune cell populations (Figure 1c,d; Figure S1d,e, Supporting Information), and these populations tend to be involved in the activation of the immune response, including CD4 T cells (CD4 T; *Rs* = −0.71; p = 4.4 × 10^−20^), CD8 T cells (CD8 T; *Rs* = −0.31; p = 4.5 × 10^−4^), and NK T cells (NKT; *Rs* = −0.31; p = 5.7 × 10^−4^). The hypoxia score positively correlated with the abundance of seven immune cell populations (Figure 1c,d; Figure S1d,e, Supporting Information), which are associated with immune escape, including natural regulatory T cells (nTreg; *Rs* = 0.55; p = 7.6 × 10^−11^), type 1 regulatory cells (Tr1; *Rs* = 0.23; p = 0.01), exhausted T cells (Tex; *Rs* = 0.18; p = 0.047) and macrophages (*Rs* = 0.52; p = 1.2 × 10^−9^). A previous study showed the activation of immune escape through inhibitory immune checkpoints^[^ ^26^ ^]^ and we further calculated the correlation between the hypoxia score and expression of 20 known inhibitory immune checkpoints^[^ ^29^ ^]^ We found the hypoxia score to be positively correlated with most immune checkpoints, including CD276 (*Rs* = 0.55; p = 3.46 × 10^−11^), PVR (*Rs* = 0.50; p = 3.40 × 10^−9^), and CD274 (PD‐L1; *Rs* = 0.42; p = 1.67 × 10^−16^) (Figure 1e). Taken together, our results suggest that hypoxia is associated with immune escape in carcinogenesis.

To dissect the crosstalk of hypoxia and the infiltration of different types of immune cells across different cancer types, we assessed the correlation between the hypoxia score and the abundance of the above 24 immune cell populations across 32 solid tumors from The Cancer Genome Atlas^[^ ^30^ ^]^ (Table S2 and Figure S2a, Supporting Information). We observed that hypoxia status positively correlated with inhibitory immune cell populations (Figure S2a, Supporting Information). For example, the hypoxia score is positively correlated with the abundance of macrophages in 19 cancer types and with the abundance of Tex in 14 cancer types. In contrast, hypoxia status negatively correlated with stimulatory immune cell populations. For example, the hypoxia score is negatively correlated with the abundance of CD4 T cells in 27 cancer types and with central memory T cells (Tcm) in 20 cancer types. This result suggests that the hypoxic TME may increase the infiltration of inhibitory immune cells and reduce the infiltration of effective immune cells. To systematically assess tumor immunogenicity, we further calculated the immunophenoscore (IPS), developed in a previous study, to present the overall score for immune response^[^ ^31^ ^]^ based on the expression of the gene sets or representative genes for four immune categories, including effector cells, suppressor cells, major histocompatibility complex (MHC) molecules, and immunomodulators (see Methods). We observed that IPSs were significantly negatively correlated with the hypoxia score (Figure 1f).

To further understand the effect of hypoxia on the efficacy of immunotherapy, we obtained RNA‐seq and scRNA‐seq data with ICB treatment response (Table S3, Supporting Information). We examined the hypoxia score between non‐responders (NR), defined as progressive disease (PD), and responders, defined as partial/complete responders (PR/CR), in two skin cutaneous melanoma (SKCM) patient cohorts with anti‐PD‐1 treatment^[^ ^32^, ^33^ ^]^ and a bladder cancer patient cohort with anti‐PD‐L1 treatment^[^ ^34^ ^]^ observing that the hypoxia score is significantly lower in responders (Figure 1g). Meanwhile, the high hypoxia score group experienced unfavorable overall survival (Figure 1h). Next, we calculated the alteration of the hypoxia score from on‐treatment to pre‐treatment and compared the difference in the hypoxia score between non‐responders and responders. We found that the hypoxia score tended to be down‐regulated in responders, while being up‐regulated in non‐responders (Figure 1i; p = 0.015). Tumor‐infiltrating lymphocytes (TILs) were negatively correlated with the hypoxia score in both pre‐treatment (Figure S2b, Supporting Information; *Rs* = −0.36) and on‐treatment (Figure S2c, Supporting Information; *Rs* = −0.51) samples. We further found that hypoxia score alteration negatively correlated with the abundance of CD4^+^ T cells (Figure S2d,e, Supporting Information; *Rs* = −0.58; p = 4.8 × 10^−5^) and positively correlated with the abundance of nTreg (Figure S2d,f, Supporting Information; *Rs* = 0.31; p = 0.048), which was associated with immune escape. The number of TCR β‐chain complementarity determining regions (CDR3s) also showed negative correlation with the hypoxia score in both pre‐treatment (Figure S2g, Supporting Information; *Rs* = −0.36, p = 0.06) and on‐treatment (Figure S2h, Supporting Information; *Rs* = −0.47, p = 0.013) samples. We further examined the effect of hypoxia on the efficacy of immunotherapy at single‐cell resolution. NR had higher hypoxia scores in T cells than responders in both pre‐ and on‐treatment samples at single‐cell resolution (Figure 1j,k). Taken together, the integration of bulk, scRNA‐seq, and spatial transcriptomics reveals that hypoxia status is associated with suppression of the immune microenvironment in tumorigenesis and resistance to anti‐PD‐1 therapy.

### 2.2 High Hypoxia Status Around Tumor Boundary Recruits *ALCAM* ^high^ Macrophages

**Figure 2:**
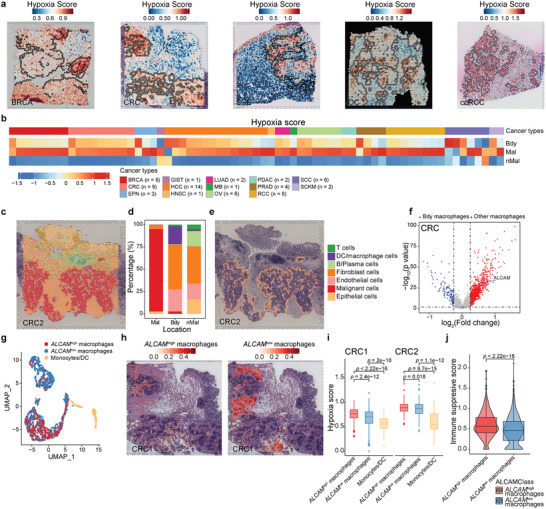
Hypoxia status around tumor boundary recruits *ALCAM* ^high^ macrophages. a) Spatial feature plots of signature scores of hypoxia status in breast cancer (BRCA), colorectal cancer (CRC), squamous cell carcinoma (SCC), ovarian cancer (OC), and clear cell renal cell carcinoma (ccRCC). A spot with a black or white circle indicates the location of the tumor boundary. b) Heatmap showing the normalized hypoxia scores in each spatial region of each sample, including BRCA (n = 8), CRC (n = 9), ependymoma (EPN, n = 3), gastrointestinal stromal tumor (GIST, n = 1), hepatocellular carcinoma (HCC, n = 14), head and neck squamous cell carcinoma (HNSC, n = 1), lung adenocarcinoma (n = 2), metastatic brain tumor (MB, n = 1), OC (n = 6), pancreatic ductal adenocarcinoma (PDAC, n = 2), prostate adenocarcinoma (PRAD, n = 4), ccRCC, n = 8, squamous cell carcinoma (SCC, n = 6), and skin Cutaneous Melanoma (SCKM n = 2). c) Spatial scatter pie plots representing the proportions of the seven cell types predicted by Cottrazm in whole CRC ST slide spots. d) Bar plots representing the proportions of the seven cell types predicted by Cottrazm in spots from malignant, tumor boundary, and non‐malignant regions. e) Spatial scatter pie plots representing the proportions of the seven cell types predicted by Cottrazm in tumor boundary spots. The colors in (c–e) indicate the cell types. f) Volcano plot exhibiting the differentially expressed genes from the macrophage tumor boundary and other remaining regions combined Bdy macrophages and other macrophages from three CRC samples (n = 3). g) The characteristics of myeloid subtypes in the ST dataset. The UMAP projections of subtypes of myeloid cells in CRC, including *ALCAM* ^high^ macrophage, *ALCAM* ^low^ macrophage, and monocyte/DC. h) Predicted proportion within each capture spot for *ALCAM* ^high^ macrophage and *ALCAM* ^low^ macrophage in CRC. i) Boxplots showing the hypoxia score among *ALCAM* ^high^ macrophages (red), *ALCAM* ^low^ macrophages (blue), and monocyte/DC (orange) in two CRC ST slides. j) The immune suppressive scores of *ALCAM* ^high^ and *ALCAM* ^low^ macrophages in CRC samples (n = 3). The boxes in (i,j) showed the median ±1 quartile, with the whiskers extending from the hinge to the smallest or largest value within 1.5× the IQR from the box boundaries. A two‐sided Wilcoxon signed‐rank test was used to assess the statistical significance in (i,j).

The hypoxia status of tumor cells were well described in previous studies,^[^ ^25^, ^35^, ^36^, ^37^ ^]^ but the hypoxia heterogeneity landscape in the tumor spatial microenvironment (TSME) has yet to be fully presented. Particularly, as the tumor boundary connects malignant and non‐malignant cells, it represents a highly heterogeneous region involving the interaction of cancer cells with various cell types, including immune and stromal cells. Here, based on our developed tool *Cottrazm* ^[^ ^38^ ^]^ we delineated the tumor boundary (black circle in **Figure** **2**a), connecting points of malignant and non‐malignant cells in tumor tissue and assessed the spatial distribution of the hypoxia score for five cancer types, including breast cancers (BRCA), colorectal cancer (CRC)^[^ ^39^ ^]^ squamous cell carcinoma^[^ ^40^ ^]^ (SCC), ovarian cancer (OC), and clear cell renal cell carcinoma^[^ ^41^ ^]^ (ccRCC). We further extended our analysis of spatial distribution of hypoxia score  to 67 slides with clear tumor boundary across 14 cancer types, we  found that in the majority of samples, the malignant spots had the highest hypoxia score, while boundary spots had significantly higher hypoxia scores than the non‐malignant spots (Figure 2a,b), suggesting hypoxic characteristics around the tumor boundary. To assess the association of hypoxia status with cellular composition in TSME, we then deconvoluted the cellular composition of CRC tissues in these three regions by *Cottrazm* ^[^ ^38^ ^]^ and compared different compositions among the malignant, tumor boundary, and non‐malignant region (Figure 2c–e). We calculated the cellular composition of 67 ST slides from 14 cancer types and we took CRC and BRAC tissue as examples to visualize the cellular composition in the entire ST slides. Our analysis indicated a predominant infiltration of macrophages in the tumor boundary compared to both malignant and non‐malignant regions in most ST slides (Figure 2c–e; Figure S3a–c, Supporting Information). This result suggests that macrophage enriched in the tumor boundary is an important characteristic in TME among cancer types, which consistent with our previous results.^[^ ^39^, ^42^ ^]^ We further found that *ALCAM* was highly expressed in the macrophages enriched in the tumor boundary (Figure 2f, Figure S3d, Supporting Information). *ALCAM* was reported contributing to the migration of macrophages and DCs. *ALCAM* macrophages were related to T cell proliferation^[^ ^43^ ^]^ and T cell‐mediated responses through *ALCAM*‐*CD6* interaction^[^ ^44^ ^]^ We further annotated the cell subtypes of DC/macrophages and divided them into three subtypes in CRC tissue: *ALCAM* ^high^ macrophages, *ALCAM* ^low^ macrophages, and DC/monocytes (Figure 2g). Compared with malignant and non‐malignant regions, *ALCAM* ^high^ macrophages were enriched in the tumor boundary, while *ALCAM* ^low^ macrophages tended to be enriched in non‐malignant regions and Mono/DC cells tended to be enriched in the region surrounding the tertiary lymphoid structure (TLS; Figure 2h; Figure S3e, Supporting Information). *ALCAM* ^high^ macrophages had the highest hypoxia score among the subtypes (Figure 2i). In addition, we identified that *ALCAM* ^high^ macrophages with higher hypoxia scores were enriched in the tumor boundary in BRCA (Figure S3f,g, Supporting Information). Furthermore, the immunosuppressive function score was calculated by immune‐related signatures^[^ ^40^ ^]^ and we observed that *ALCAM* ^high^ macrophages contribute much more than *ALCAM* ^low^ macrophages to immune suppression in CRC patients (Figure 2j). To quantify the distribution of *ALCAM* ^high^ macrophages, we calculated the enrichment of *ALCAM* ^high^ macrophages across six cancer types with at least five samples, including CRC (n = 6), BRCA (n = 8), SCC (n = 6), OC (n = 6), RCC (n = 5), and HCC (n = 14). We found *ALCAM* ^high^ macrophages significantly enriched or have the enrichment tendency in the tumor boundary (Figure S3h, Supporting Information). These results suggest that the hypoxic state around the tumor boundary may recruit *ALCAM* ^high^ macrophages.

### 2.3 Hypoxia Increases *ALCAM* Expression in Macrophages Through HIF1A Regulation

**Figure 3:**
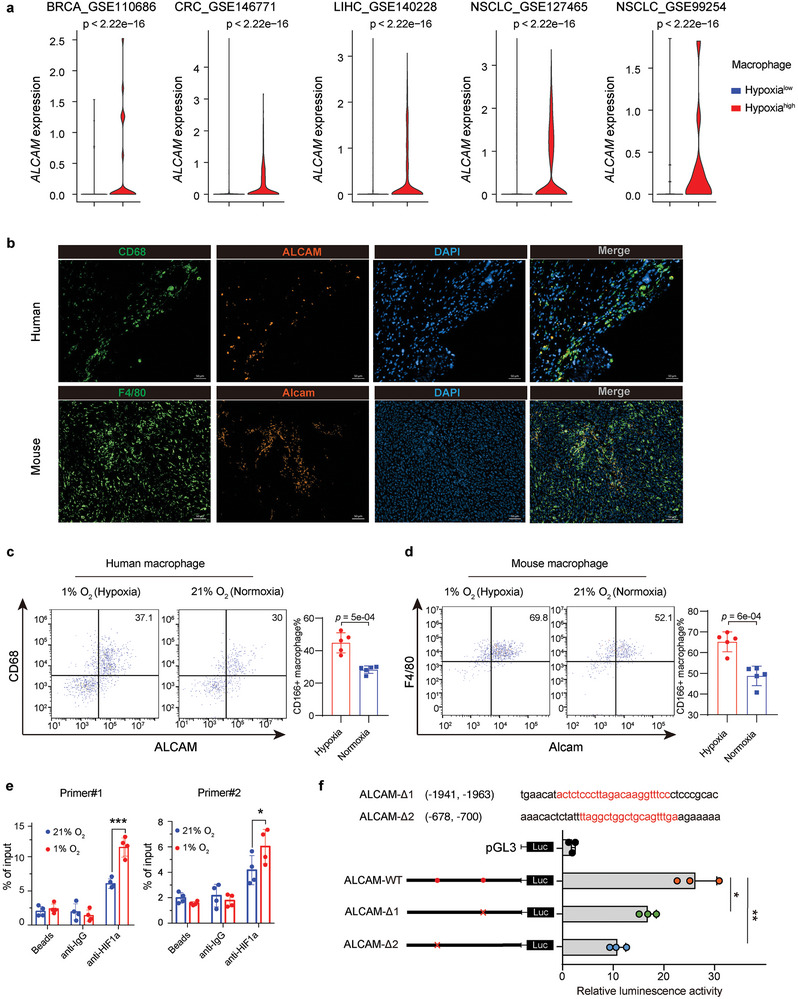
Hypoxia increases *ALCAM* expression in macrophages through HIF1A. a) Violin plot showing RNA expression of ALCAM in macrophages with hypoxia score‐high and ‐low groups in five independent single‐cell transcriptomics datasets. b) Representative mIHC images indicated ALCAM is expressed on macrophages in human and mouse tumor tissues. Scale bars, 50 µm. c,d) Flow cytometry analyzing the expression of ALCAM on primary human (c) and mouse (d) macrophages under hypoxic stimulation. e,f) ChIP‐qPCR (e) and Luciferase reporter assay (f) validated that the binding site of two predicted binding regions in Alcam promoter under 21% O_2_ and 1% O_2_ condition. The boxes in (f) show the median ±1 quartile, with the whiskers extending from the hinge to the smallest or largest value within 1.5× the IQR from the box boundaries. Statistical analysis was performed by two‐side Wilcoxon signed‐rank test in (a,e,f). Statistical analysis was performed by unpaired *t*‐test in (c,d).

To investigate the regulation of hypoxia in macrophages with *ALCAM* expression, we evaluated the hypoxia score in macrophages at single‐cell resolution and stratified the macrophages into hypoxia score high (hypoxia^high^) and low (hypoxia^low^) groups based on the median expression of the hypoxia score in multiple scRNA‐seq datasets obtained from TISCH^[^ ^45^ ^]^ database. We found *ALCAM* to be significantly expressed in hypoxia^high^ macrophages (**Figure** **3**a). We then validated that *ALCAM* is expressed on macrophages in human and mouse tumor tissues (Figure 3b). Then, flow cytometry and western blot experiments revealed increased expression of ALCAM on macrophages under hypoxic stimulation (Figure 3c,d; Figure S4a, Supporting Information). When knocked down (KD) or inhibited Hif1a in macrophage, Alcam significantly decreased under hypoxic condition (Figure S4b,c, Supporting Information). Since Hif1a is a master transcriptional regulator of cellular response to hypoxia, we hypothesized that Hif1a can bind to the promoter region of *ALCAM* to regulate its expression. We predicted two potential Hif1a binding regions and performed chromatin immunoprecipitation‐qPCR (ChIP‐qPCR) under hypoxic and normoxic conditions. We validated that Hif1a can bind to the promoter of *ALCAM* and enhance the binding efficacy under hypoxic conditions (Figure 3e). To confirm that the Hif1a binding region contributes to downstream gene expression, we performed the luciferase reporter assay to validate that Hif1a can bind to the promoter of Alcam and enhance the binding efficacy under hypoxic conditions (Figure 3f). These results suggest that hypoxia increases *ALCAM* expression in macrophages through HIF1Ai binding at the *ALCAM* promoter.

### 2.4 Hypoxia Is Associated With Both Exhausted CD8^+^ T and CD4^+^ T Cell Differentiation in Cancers

**Figure 4:**
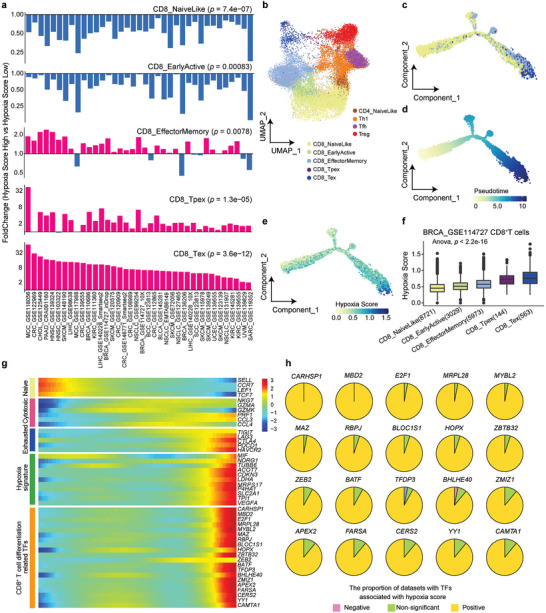
Association between hypoxia status and CD8^+^ T cell differentiation. a) Bar plot showing the fold change for CD8^+^ T cell subset proportion between hypoxia score‐high and ‐low groups in 38 single‐cell transcriptomics datasets. b) UMAP plot showing subclusters of breast cancer (BRCA) CD8^+^ T cells and CD4^+^ T cells, includingCD8_NaiveLike, CD8_EarlyActiv, CD8_EffectorMemory, CD8_Tpex, CD8_Tex, CD4_NavieLike, Th1, Tfh and Treg. c) Differentiation trajectory of CD8^+^ T cell subsets inferred by Monocle2. d) BRCA CD8^+^ T cells ordered by pseudotime. e) Dynamic alteration of hypoxia score during BRCA CD8^+^ T cell differentiation. f) Box plot with hypoxia score for CD8^+^ T cell subsets in an increasing order. g) Heatmap of gene differential expression along with CD8^+^ T cell differentiation. h) Pie plot showing the proportion of datasets in which TFs are positively, negatively, or non‐significantly related with hypoxia score. The boxes in (f) show the median ±1 quartile, with the whiskers extending from the hinge to the smallest or largest value within 1.5× the IQR from the box boundaries. A two‐sided Wilcoxon signed‐rank in (a) and a one‐way ANOVA test was performed in (f).

The exhaustion of T cells is significantly associated with hypoxia (Figure 1c,d). To further explore the relationship of hypoxia and immunosuppressive cells with exhausted T cells, we collected publicly available scRNA‐seq data that include 38 CD8^+^ T cell datasets (Table S4, Supporting Information). After embedding these scRNA‐seq data into a T cell reference atlas, we divided CD8^+^ T cells into five subpopulations: CD8_NaiveLike, CD8_EarlyActiv, CD8_EffectorMemory, CD8_Tpex and CD8_Tex^[^ ^46^ ^]^ We grouped CD8^+^ T cells into hypoxia score‐high and ‐low groups and compared the composition of T cell subsets between these two groups. We found T cells with high hypoxia score have significantly higher proportions in both CD8 Tex and CD8 Tpex than those T cells with low hypoxia score in all CD8^+^ T cell datasets (**Figure** **4**a), which suggests that CD8^+^ T cells gradually present exhausted phenotypes under hypoxia.

To explore the association between hypoxia status and CD8^+^ T differentiation, we performed trajectory analysis of CD8^+^ T cells to place all cells along a trajectory corresponding to cell differentiation (see Methods, Figure 4b,c)^[^ ^47^ ^]^ T cells developed from a naïve state to an exhausted state in the TME (Figure 4c,d). The hypoxia score is progressively increased with the trajectory of CD8^+^ T cell differentiation (Figure 4c–f). In addition, we noticed naïve CD8^+^ T cell‐related regulators (e.g., *SELL, TCF7*) were down‐regulated in this differentiation process while exhausted CD8^+^ T cell‐related regulators (e.g., *TIGIT*, *LAG3*, *CTLA4*, *PDCD1*, *HAVCR2*) and hypoxia signatures (e.g., *LDHA*, *VEGFA*, *SLC2A*) were upregulated (Figure 4g). To explore potential transcription factors (TFs) driving CD8^+^ T cell differentiation induced by hypoxia, we obtained 20 TFs whose expression gradually increased along with the differentiation process (Figure 4g). Most TFs are enriched with functions in T cell development. For example, MBD2 abrogation causes impaired induction of memory CD8^+^ T cells^[^ ^48^ ^]^ RBPJ‐dependent Notch signaling plays an important role during T cell differentiation^[^ ^49^ ^]^ Meanwhile, we observed that these TFs were positively correlated with hypoxia scores in most of the datasets (Figure 4h), suggesting a potential regulatory role of hypoxia on these TFs.

Similarly, to perform a parallel analysis of CD8^+^ T cells, we divided CD4^+^ T cells into four subpopulations, CD4_NavieLike, Th1, Tfh, and Treg, in 34 CD4^+^ T cell datasets and found suppressive immune cells at the end of differentiation, including Tfh and Treg, to be enriched in the hypoxia score‐high group (Figure S5a, Supporting Information). The hypoxia score is progressively increased with the trajectory of CD4^+^ T cell differentiation in cancer patients (Figure S5b–e, Supporting Information). The hypoxia‐related top 20 TFs also showed an increasing trend during the cell differentiation trajectory (Figure S5f,g, Supporting Information).

We also collected mouse scRNA‐seq data to verify whether the function of hypoxia in mice tumors is conserved for subsequent experimental validation. The result in colon cancer or melanoma bearing mice showed high degrees of similarity with that in humans (Figures S6 and S7a–e, Supporting Information). Meanwhile, the majority of hypoxia marker genes, such as *ALDOA*, *ENO1*, *PGAM1*, and *TPI1*, showed a significant increasing tendency during the differentiation of CD4^+^ T cells (Figure S7f, Supporting Information). All these results suggest that the hypoxic tumor environment may be associated with the T cell differentiation in the exhaustion status and is associated with the immunosuppressive microenvironment, thus, inhibiting the efficacy of ICB treatment.

### 2.5 *ALCAM* ^high^ Macrophages Associated With the Exhaustion of T Cells Around the Tumor Boundary

**Figure 5:**
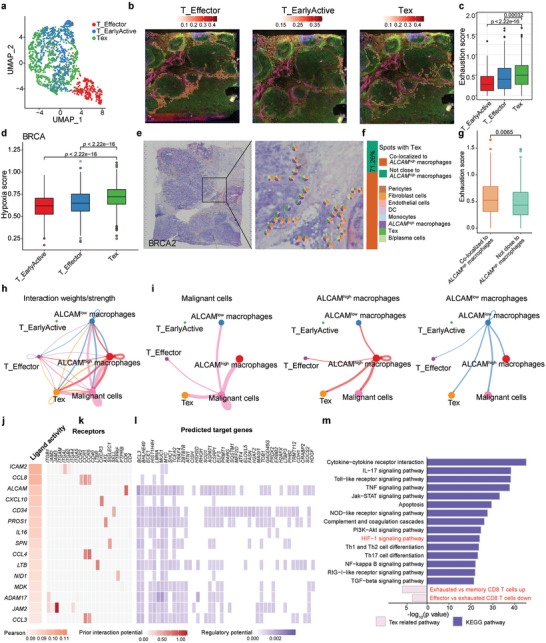
*ALCAM* ^high^ macrophage crosstalk with exhausted CD8 T cells in tumor spatial microenvironment, a) The characteristics of T cell subtypes in the ST dataset. The UMAP projections of subtypes of myeloid cells in BRCA, including T_Effector, T_EarlyActive, and Tex (exhausted CD8 T cells). b) Predicted proportion within each capture spot for T_Effector, T_EarlyActive, and Tex in BRCA. c,d) The exhaustion score (c) and hypoxia score (d) among T_Effector, T_EarlyActive, and Tex. e–g) Spatial scatter pie plots (e) showing the proportions of the eight cell types for spots with co‐localization of *ALCAM* ^high^ macrophages and Tex; and bar plots (f) showing proportion of Tex spots colocalized with or without *ALCAM* ^high^ macrophages. (g) Box plots showing the exhaustion score between Tex spots colocalized with or without *ALCAM* ^high^ macrophages. h,i) Circle plot visualizing the cellular communication of cell types among malignant cells, *ALCAM* ^high^ macrophages, *ALCAM* ^low^ macrophages, T_Effector, T_EarlyActive, and Tex cells. Circle sizes are proportional to the number of cells in each cell group, and thickness of the flow represents the interaction weight. j) Top‐ranked ligands inferred to regulate exhausted *CD8* ^+^ T cells by *ALCAM* ^high^ macrophages by NicheNet. k) Ligand‐receptor pairs showing interaction between *ALCAM* ^high^ macrophages and exhausted *CD8* ^+^ T cells ordered by ligand activity (j). l) Heatmap showing regulatory potential of top 15 ranked ligands (j) and the downstream target genes in exhausted *CD8* ^+^ T cells. m) Representative KEGG pathways and related exhausted pathway enrichment of the predicted target genes expressed in exhausted *CD8* ^+^ T cells. The boxes in (c,d,g) show the median ±1 quartile, with the whiskers extending from the hinge to the smallest or largest value within 1.5× the IQR from the box boundaries. A two‐sided Wilcoxon signed‐rank test was used to assess statistical significance in (c,d,g), Fisher's test in (m).

To further explore the association of hypoxia status and T cell status, based on sub‐spot gene expression profiles reconstructed by Cottrazm^[^ ^38^ ^]^ we performed unsupervised graph‐based clustering and used marker‐based annotation to define the cell types. We annotated the cell subtypes of T cells and divided them into three subtypes in BRCA tissue: T_EarlyActive, T_Effector, and Tex (exhausted CD8 T cells). Compared with malignant and non‐malignant regions, Tex was enriched in the tumor boundary, while T_Effector and T_EarlyActive tended to be enriched in non‐malignant regions (**Figure** **5**a,b). Tex had the highest exhaustion score and hypoxia score among these three T cell subtypes (Figure 5c,d). Furthermore, there are three T cell subtypes identified in CRC TSME (Figure S8a,b, Supporting Information). Tex in CRC has higher exhaustion signature and hypoxia score and is localized around the tumor boundary (Figure S8c, Supporting Information), which is consistent with T subtypes in BRCA TSME.

Both *ALCAM* ^high^ macrophages and Tex tend to be enriched at the tumor boundary and hypoxia can induce the expression of *ALCAM* in macrophages. We therefore investigated the localization between these two cell types and other cell types. We compared the co‐localization of *ALCAM* ^high^ macrophages with T cell sub‐clusters (Tex, T_Effector, T_EarlyActive) and other cell types. We also calculated the co‐localization proportion of *ALCAM* ^low^ macropahges and Tex. We found that a high proportion of spots with Tex infiltration are co‐localized with *ALCAM* ^high^ macrophages (Figure 5e,f; Figure S8d,e, Supporting Information). Further, Tex co‐localized to *ALCAM* ^high^ macrophages showed significantly higher exhaustion scores than those far away from *ALCAM* ^high^ macrophages (Figure 5g), Tex showed high cell interaction with hypoxia^high^ macrophages or *ALCAM* ^high^ macrophages but not hypoxia^low^ macrophages or *ALCAM* ^low^ macrophages (Figure 5h,i; Figure S8f, Supporting Information), suggesting that the colocalization of *ALCAM* ^high^ macrophages and Tex may be associated with the exhaustion of T cells. Cell‐to‐cell interaction analysis showed strong interaction among hypoxia^high^ or *ALCAM* ^high^ macrophages, Tex cells, and malignant cells (Figure 5h,i; Figure S8g, Supporting Information). In addition to the macrophage migration inhibitory factor (MIF) signaling pathway, which is a well‐known HIF‐1α‐dependent pathway^[^ ^50^ ^]^ we found the *ALCAM* signaling pathway network presented between hypoxia^high^ or *ALCAM* ^high^ macrophages and Tex (Figure S8g, Supporting Information). Therefore, we investigated whether *ALCAM* ^high^ macrophages may facilitate the exhaustion of T cells. The cell interaction between *ALCAM* ^high^ macrophages and Tex revealed by NicheNet analysis^[^ ^51^ ^]^ showed high *ICAM2*, *CCL8*, and *ALCAM* ligand activity (Figure 5j), which interacted with receptors on Tex, resulting in the expression of target genes that are enriched in T cell exhaustion‐related pathways, including *BCL3*, *CISH*, *CSRP1*, *APP*, and TF *BHLHE40* (Figure 5j–l). These TFs were positively correlated with the hypoxia score in our scRNA‐seq analysis (Figure 4h). In addition, these target genes were also highly enriched in pathways of cytokine‐cytokine receptor interaction, HIF‐1 signaling pathway, and exhausted related pathways in immunologic signature gene sets (Figure 5m). Taken together, our findings suggest that hypoxia promoted *ALCAM* ^high^ macrophages interacted with Tex to promote T cell exhaustion.

### 2.6 Inhibition of Hypoxic State Reduces *ALCAM* ^high^ Macrophages and Exhausted CD8^+^ T Cells to Enhance the Therapeutic Efficacy of PD‐1 Blockade

**Figure 6:**
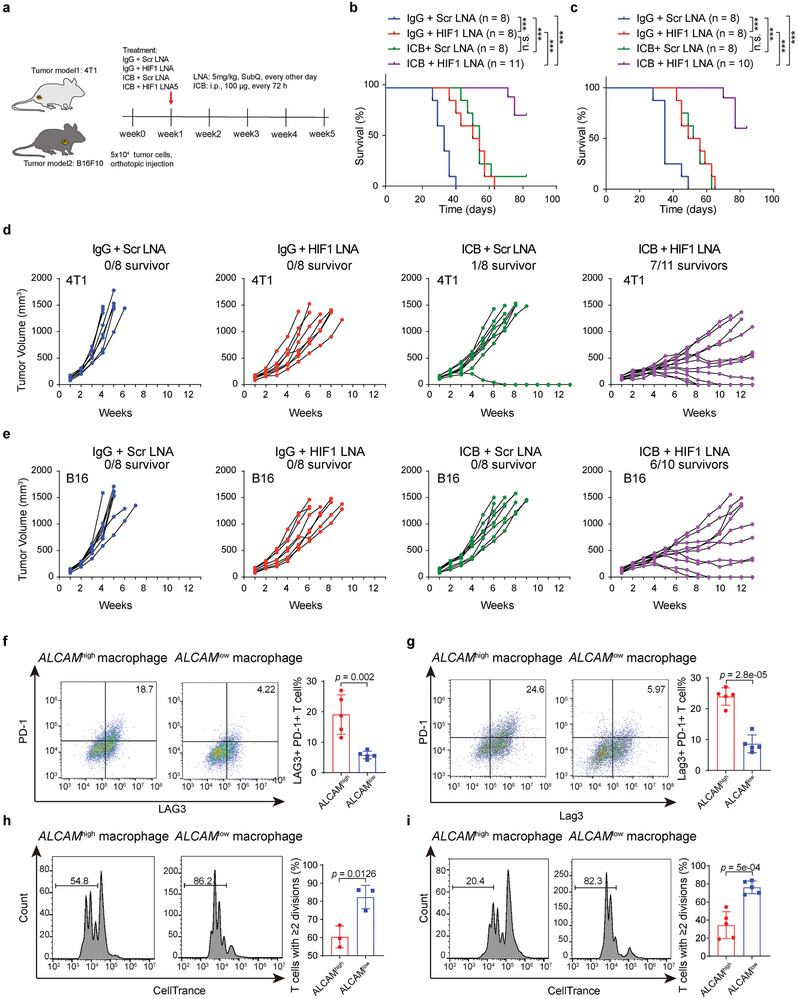
Synergistic therapeutic effect of anti‐HIF1 combined with PD‐1 blockade, a) Graphic illustration of syngeneic models established from 4T1 and B16F10 cells for combinatorial treatment. b,c) Kaplan–Meier survival analysis of syngeneic 4T1 mice model treated with scramble or HIF1 LNA (treated with EZN‐2968), immune checkpoint blocks (ICB) alone or in combination (n = 8, 8, 8, 11 animals) (b), log rank test. (c) Kaplan–Meier survival analysis of syngeneic B16F10 mice model treated with scramble or HIF1 LNA, immune checkpoint blocks (ICB) alone or in combination (n = 8, 8, 8, 10 animals). d,e) Tumor volumes of syngeneic 4T1 (d) and B16F10 (e) mice model treated with scramble or HIF1 LNA, ICB alone or in combination. f,g) Flow cytometry analyzing the expression of PD‐1 and Lag3 in *ALCAM* ^high^ and *ALCAM* ^low^ macrophages from human (f) and mouse tumor tissues (g). h,i) CellTrace dilution assay to assess the division of CD8 T cell co‐cultured with *ALCAM* ^high^ and *ALCAM* ^low^ macrophages from human (h) and mouse tumor tissues (i). A two‐sided unpaired *t*‐test was used to assess statistical significance in (f–i).

To overcome the hypoxia effect on immunotherapy, we developed the syngeneic mouse tumor model using 4T1 and B16F10 cells and treated them in different treatment strategies (**Figure** **6**a). In brief, 6‐week‐old female BALB/cJ mice were injected with 4T1 cells into the right inguinal fat pad (5 × 10^4^), or 8‐week‐old female C57BL/6 mice were injected subcutaneously with B16F10 tumor cells (5 × 10^4^). Afterward, mice with tumors reaching ≈100 mm^3^ in size were randomized into four groups with different treatment strategies: scramble (IgG), treatment with anti‐PD‐1 antibody, treatment with *HIF1* LNA (EZN‐2968), and the combination treatment of anti‐PD‐1 and *HIF1* LNA. Treatments with anti‐PD‐1 (ICB) were administered every 3 days and treatments with *HIF1* LNA were administered every other day until tumor capture, and tumor growth was measured every week. *HIF1* LNA alone substantially extended the overall survival time (median survival: 50 versus 33 days; *p* < 0.001, log‐rank test) of 4T1 tumor‐bearing mice, and combined with ICB, enhanced the survival benefit (median survival: > 91 versus 33 days; *p* < 0.0001; Figure 6b). The B16F10 model showed similar results (Figure 6c). In the 4T1 or B16F10 tumor‐bearing mice decreased tumor growth after *HIF1* LNA or ICB treatment, while a combination treatment of *HIF1* LNA and ICB achieved better efficacy (Figure 6d,e). To verify the function of *ALCAM* ^high^ macrophages, we isolated *ALCAM* ^high^ and *ALCAM* ^low^ macrophages from human and mouse tumor tissues and then co‐cultured them with CD8 T‐cells. We found that compared to *ALCAM* ^low^ macrophages, *ALCAM* ^high^ macrophages increased the expression of CD8 T‐cell exhaustion genes PD‐1 and Lag3 (Figure 6f,g; Figure S9a,b, Supporting Information). Next, we examined the proliferative ability of the T cells co‐cultured with *ALCAM* ^high^ or *ALCAM* ^low^ macrophages using the CellTrace labeling and dilution assay and found that T cells co‐cultured with *ALCAM* ^high^ macrophages proliferated much less than with *ALCAM* ^low^ macrophages compared to *ALCAM* ^low^ macrophages (Figure 6h,i). To validate the alteration of immune features in the enhanced therapeutic efficacy of *HIF1* LNA, we investigated the tumor‐immune microenvironment of B16F10 tumor‐bearing mice treated with *HIF1* LNA and ICB in combination or alone. Then, the mIHC analysis showed that *HIF1* LNA significantly reduced HIF1α expression, *ALCAM* expression, and the infiltration of macrophages and TIM3^+^ CD8^+^ T cells, and the combination treatment enhanced this efficacy (Figure S9c, Supporting Information). Further, mIHC analysis showed that *HIF1* LNA reduced the expression of PD‐L1 (Figure S9d,e, Supporting Information). Further, the mRNA expression level of HIF1A was found to be positively correlated with mRNA expression PD‐L1 (CD274) in a variety of cancer types in the TCGA cancer patient dataset (Figure S9f, Supporting Information). *HIF1* LNA increased the population of tumor infiltrating CD8^+^ T cells and their activity in the tumor and combination treatment enhanced this efficacy (Figure S9g,h, Supporting Information), further supporting that hypoxia status influences T cell differentiation and function. Taken together, the combination of *HIF1* LNA and ICB demonstrated significant reduction in *ALCAM* ^high^ macrophages and exhaustion CD8^+^ T cells, and improvement in tumor burden, survival rate, and cytolytic activity. Therefore, these findings suggest that inhibition of HIF1A could be associated with the reduce of tumor progression and resistance to ICB treatment. Further, hypoxia‐associated *ALCAM* ^high^ macrophages might be related to the immunosuppressive state of T cell subsets which may limit the efficacy of ICB treatment.

## 3 Discussion

The TME undergoes remodeling when adapting to a hypoxic environment^[^ ^16^ ^]^ However, dynamic alteration and remodeling during tumorigenesis is unclear, especially for non‐tumor cells, including immune cells in the TME that suffer from serious oxygen insufficiency. We integrated bulk, single‐cell, and spatial transcriptomics to dissect the regulatory roles of hypoxia in immune evasion through the *ALCAM* ^high^ macrophage‐Tex axis in single‐cell and spatial resolutions.

TAMs have been reported to stimulate tumor progression through various mechanisms, such as promoting tumor cell invasion and promoting tumor cell angiogenesis^[^ ^52^ ^]^ Hypoxic regions can attract immunosuppressive TAMs through releasing an increasing gradient of migratory stimulating factors and then fixing them in tumor compartments^[^ ^53^ ^]^ In our study, we identified that macrophage infiltration is most positively correlated with hypoxia level across 32 cancer types in TCGA and further identified that *ALCAM* ^high^ macrophages were significantly enriched around the tumor boundary under a hypoxic microenvironment. The tumor size might confound for this result, but unfortunately, all datasets in this study were obtained from public datasets without clinical information for tumor size, which prevent us to adjust the effect of tumor size. Further, we demonstrated that hypoxia can induce the expression of *ALCAM* in macrophages through the binding of HIF1A on the *ALCAM* promoter. The *ALCAM* signaling pathway is an important regulatory interaction between hypoxia score‐high macrophages and exhausted CD8^+^ T cells, consistent with specific expression of *ALCAM* in hypoxia score‐high macrophages, which suggests that hypoxia may induce *ALCAM* expression, thus interact with exhausted CD8^+^ T cells and finally lead to a suppressive immune microenvironment.

Besides macrophages, the differentiation of T cells is also affected by hypoxia. A recent study discovered that antigen stimulation under hypoxia renders T cell exhaustion in mice^[^ ^20^ ^]^ but whether this is the situation in cancer patients is unclear. Here, we demonstrated that hypoxia score consistently increased with the differentiation trajectory of both CD8^+^ T cells and CD4^+^ T cells. The hypoxia score is gradually increased from naïve T cells to effector T cells and then to the exhausted state, and hypoxia status was also significantly correlated with transcription factors that are related to T cell differentiation and exhaustion. Among these TFs, *YY1*,^[^ ^54^ ^]^ *E2F1*,^[^ ^55^ ^]^ *MAZ*,^[^ ^56^ ^]^ *ZEB2*,^[^ ^57^ ^]^ *BHLHE40*,^[^ ^58^ ^]^ and *CERS2* ^[^ ^59^ ^]^ were reported to be induced by HIF1A. Further investigation is warranted to validate whether the hypoxic tumor microenvironment drives the TF changes and T cell differentiation. Overall, these results suggest hypoxia status is associated with T cell differentiation and is highly correlated with exhausted T cells. Additionally, hypoxia related *ALCAM* ^high^ macrophages interacted with CD8^+^ T cells may promote T cell exhaustion.

Blockades targeting immune checkpoints that have been approved for the treatment of human cancers have shown considerable clinical effects^[^ ^60^ ^]^ Hypoxia induces a series of biological changes that contribute to tumorigenesis and is a critical issue that leads to resistance to a broad range of therapeutic options, including immunotherapy^[^ ^37^ ^]^ Therefore, understanding the effect of hypoxia on immune features is crucial to improving the outcomes of cancer immunotherapy. Our research revealed successively increased hypoxia status in morphological stages during carcinogenesis and identified hypoxia‐associated immune features in tumors before and after immunotherapy. Hypoxia is negatively associated with effective immune cells or immune features (e.g., TCR), and positively associated with T cell exhaustion. Hypoxia‐associated *ALCAM* ^high^ macrophages may crosstalk with T cells to regulate the exhaustion status, we compared the exhaustion scores of Tex co‐localized to *ALCAM* ^high^ macrophages versus Tex distant from *ALCAM* ^high^ macrophages, and found Tex co‐localized to *ALCAM* ^high^ macrophages showed higher exhaustion scores (Figure 5e–g). In addition, the cell‐to‐cell interactions analysis also showed strong interaction among *ALCAM* ^high^ macrophages and Tex (Figure 5h,i). To prove that the co‐localization promotes T cell exhaustion, we co‐cultured *ALCAM* ^high^ macrophages and *ALCAM* ^low^ macrophages from human or mouse CRC tumor with T cells separately, then using FACS to assess the expression levels of exhaustion markers (PD‐1 and LAG3) in T cells. The results showed that T cells co‐cultured with *ALCAM* ^low^ macrophages express higher level of exhaustion marker (PD‐1 and LAG3, Figure 6f,g). Therefore, we revealed that remodeling of the tumor immune suppressive microenvironment via the hypoxia‐*ALCAM* ^high^macrophage‐Tex axis may contribute to immunotherapy resistance. Preclinically, the inhibition of HIF1a by EZN‐2968 enhanced the therapeutic efficacy of PD‐1 blockade in the treatment of both melanoma and TNBC by reducing the infiltration of *ALCAM* ^high^ macrophages and exhausted CD8^+^ T cells and promoting anti‐tumor immunity. Three out of eleven 4T1 tumor‐bearing mice and two out of ten B16F10 tumor‐bearing mice showed complete disappearance of tumors within 9–10 weeks after combination treatment, which provides a novel strategy for EZN‐2968 and ICB therapy to enhance immunotherapy efficacy. Taken together, our work reveals the hypoxia‐based intercellular communication hub through the hypoxia‐*ALCAM* ^high^macrophage‐Tex axis in single‐cell and spatial levels that supports immune evasion. Thus, therapeutic strategy that inhibits the hypoxic state can break the *ALCAM* ^high^macrophage‐Tex axis and be combined with ICB treatment to improve the efficacy of immunotherapy.

## 4 Experimental Section

### Data Collection, Process, and Analysis—Bulk RNA‐Seq Datasets

Gene expression profiles of 122 biopsies at successive morphological stages during lung squamous carcinogenesis were obtained from Gene Expression Omnibus (https://www.ncbi.nlm.nih.gov/geo/; GSE33479). The 122 biopsies were distributed according to histology and fluorescence status as follows: 13 biopsies with normal histology and normal fluorescence, 14 with normal histology and hypo‐fluorescence, 15 with hyperplasia, 15 with metaplasia, 13 with mild dysplasia, 13 with moderate dysplasia, 12 with severe dysplasia, 13 with carcinoma in situ, and 14 with squamous cell carcinoma. The process of carcinogenesis was divided into 9 stages, ranging from stage 0 and 1, representing bronchial mucosa with normal histology, which had normal and low fluorescence, respectively, to stage 2, which was hyperplasia, stage 3, which was comprised metaplasia, stage 4 and 5, which were mild and moderate dysplasia, respectively, stage 6, which was combined severe dysplasia, stage 7, which was in situ carcinoma into “high‐grade” lesions, and stage 8, which represents segregated invasive characteristics from premalignant lesions. Gene expression profiles of biopsies at successive stages during prostate carcinogenesis were obtained from GSE6099, including 12 with benign prostatic hyperplasia, 13 with prostatic intraepithelial neoplasia (PIN), 32 with prostate carcinoma (PCA), and 20 with metastatic prostate cancer (MET). Normalized gene expression data of 32 cancer types was downloaded from TCGA data portal (https://portal.gdc.cancer.gov/).

### Spatial Transcriptomics

The prostate spatial transcriptomic data, including count matrices and images, were provided by Andrew Erickson et al. from the Mendeley database^[^ ^24^ ^]^ Spatial transcriptomics of CRC were obtained from Genome Sequence Archive with accessible ID HRA000979^[^ ^39^ ^]^ BRCA and OC spatial transcriptomic data were obtained from 10X genomics official website (https://support.10xgenomics.com spatial‐gene‐expression/datasets), ccRCC (GSE175540)^[^ ^41^ ^]^ and SCC (GSE144240)^[^ ^61^ ^]^ Spatial transcriptomics of 67 tumor tissues with covering 14 cancer types and with clear tumor boundaries were collected from the web available portal SpatialTME (https://www.spatialtme.yelab.site/)^[^ ^62^ ^]^

### Single‐Cell Transcriptomics

Thirty‐five single‐cell transcriptomics datasets with metadata, 38 CD8^+^ T cells datasets and 34 CD4^+^ T cells datasets were obtained from Tumor Immune Single‐cell Hub (TISCH)^[^ ^45^ ^]^ To analyze T cell trajectory, we obtained human single‐cell gene expression matrices from European Genome‐phenome Archive (EGA) under study no. EGAS00001004809 and no. EGAD00001006608^[^ ^63^ ^]^ For mouse T cells, we used single‐cell expression matrices collected by Massimo Andreatta et al.^[^ ^46^ ^]^ (https://github.com/carmonalab/ProjecTILs_CaseStudies).

Single‐cell data to compare hypoxia score across cell types and PD1 treatment response were processed by TISCH^[^ ^45^ ^]^ including GSE123813 and GSE120575. Molecular profiles for patients with ICB treatment were obtained from GEO, including GSE78220 and GSE91061. IMvigor210 cohort with the expression data and detailed clinical information were downloaded from http://research‐pub.gene.com/IMvigor210CoreBiologies.

### Hypoxia Score Calculation

We calculated the “hypoxia score” by using gene set variation analysis^[^ ^64^ ^]^ based on a 15‐gene signature^[^ ^65^ ^]^ which has been used in recent papers to classify the hypoxia status across multiple TCGA cancer types.^[^ ^66^, ^67^ ^]^ These 15 hypoxia related genes include *ACOT7*, *ADM* ^[^ ^68^ ^]^ *ALDOA* ^[^ ^69^ ^]^ *CDKN3*, *ENO1* ^[^ ^70^ ^]^ *LDHA* ^[^ ^71^ ^]^ *MIF* ^[^ ^72^ ^]^ *MRPS17*, *NDRG1* ^[^ ^73^ ^]^ *P4HA1* ^[^ ^74^ ^]^ *PGAM1* ^[^ ^75^ ^]^ *SLC2A1* ^[^ ^76^ ^]^ *TPI1*, *TUBB6*, and *VEGFA* ^[^ ^77^ ^]^ all of which associated with HIF1A as described in previous studies. Furthermore, a recent study systematically assessed the robustness of different hypoxia signatures and suggested that the hypoxia signature we used was the best performing signature^[^ ^78^ ^]^ Scoring hypoxia scores of single‐cell data was conducted as previously described^[^ ^79^ ^]^ (https://www.github.com/cssmillie/ulcerative_colitis). In brief, the gene signature score for each cell was calculated by the mean scaled expression across all genes in the signatures.

### Spatial Transcriptomics Data Analysis for 10X Genomics ST Platform “1k”

Visualizing and analyzing spatial transcriptomics data of prostate cancer was generated with R package *STutility* ^[^ ^80^ ^]^ These ten cubes were all sequenced by 10X genomics ST platform “1k”. The data were loaded and underwent filtering, including (1) keeping genes that were found in at least 5 capture spots and have a total count value > = 100; (2) keeping the capture‐spots that contain > = 500 total transcripts. Next, we projected the hypoxia score on them with *ST.FeaturePlot*.

### Definition of Tumor Boundary‐Based ST Data

The malignant (Mal) region, tumor boundary (Bdy), and non‐malignant (nMal) regions of the ST slides (BRCA, n = 2; CRC, n = 2; OC, n = 1; and ccRCC, n = 1) were defined by the method as previous study^[^ ^38^ ^]^ shown. Malignant regions were the ST spots occupy malignant cells while tumor boundary was spots connecting malignant and non‐malignant cell spots in tumor tissues. “BoundaryDefine” function was used in *Cottrazm* package^38^to delineate the tumor boundary. Initially, based on neighboring spot information and morphological distances of HE‐staining images, Cottrazm normalizes ST gene expression data to obtain a morphologically adjusted expression matrix. Then clustered by k‐nearest neighbor (KNN) algorithm, clusters were identified using copy number variation by InferCNV which define the core spots of malignant cells. Further, Cottrazm arranged spatial spots on hexagonal systems, extrapolated layer by layer from core spots of malignant cells and determined the identity of spots according to UMAP distance to tumor centroid as Malspots or Bdy spots. When all neighbors of Mal spots were not classified as tumor tissue, the extrapolation was completed. Remaining spots were therefore labeled as nMal spots. The boundary macrophage‐specific genes were obtained by “FindDiffGenes” function of the *Cottrazm* package.

### Deconvolution and Reconstruction of ST Spots

The cellular composition of spatial spots was calculated using the “SpatialDecon” function, and pie plots and bar plots of deconvolution were generated with “DeconPieplot” and “DeconBarplot” functions in the *Cottrazm* package. The reconstruction results at sub‐spot levels of CRC and BRCA slides were obtained by the “SpatialDecon” function of the *Cottrazm* package, then the *Seurat* package was used to normalize, scale, and obtain sub‐clusters of myeloid cells and T cells. Subsequently, based on the reconstruction result, we filtered macrophages and T cells. In the reconstruction result of macrophages, we divided macrophages in BRCA into *ALCAM* ^high^ macrophages and *ALCAM* ^low^ macrophages based on the median value of normalized *ALCAM* expression; in CRC data, we first defined *ALCAM* ^high^ macrophages based on normalized *ALCAM* expression, then define Mono/DC based on differential expression genes. In the reconstruction result of T cells, we divided T cell subsets based on signature genes, for example *PDCD1*, *LAG3*, *HAVCR2*, *CD8A*, *CD8B*, *CD3E*, *ENTPD1*, *TGAE*, *BATF*, *NR4A1* for Tex. The function “find_neighbors” in the *Cottrazm* package was used to identify the first outer circle of co‐localized spots of Tex and *ALCAM* ^high^ macrophages.

### Cell‐To‐Cell Communication Analysis

First, we projected *ALCAM* ^high^ macrophages and Tex to spatial based on barcodes, when a spatial spot contains *ALCAM* ^high^ macrophages and Tex simultaneously, the spot was denoted as *ALCAM* ^high^ macrophages and Tex co‐localization. Then, CellChat^[^ ^51^ ^]^ (version 1.1.1) was used to analyze cell‐to‐cell communication. First, a cellchat object was created by grouping in defined clusters. The ligand‐receptor interaction database we used for analysis was “CellChatDB.human” without additional supplement. Preprocessing steps were all conducted with default parameters. Then, *computeCommunProb* and *computeCommunProbPathway* were performed to infer the network of each ligand‐receptor pair and each signaling pathway separately. A hierarchy plot, circle plot and heatmap were used as different visualization forms.

NicheNet^[^ ^51^ ^]^ was used to infer the mechanisms of interaction between macrophages and T cells of the reconstruction results. For ligand and receptor interactions, clustered cells with gene expression over 10% were considered. The top 300 ligands and top 2000 targets of differentially expressed genes of “sender cells” and “affected cells” were extracted for paired ligand‐receptor activity analysis. When evaluating the regulatory network of macrophages on T cells, Tex was considered as the receiver cells and other T cell sub‐cluster cells were used as reference cells to check the regulatory potential of *ALCAM* ^high^ macrophages on Tex.

### Analysis of Immune Cell Infiltration

The abundance of 18 T‐cell subsets (CD4^+^ naïve, CD4^+^ T, CD8^+^ naïve, CD8^+^ T, central memory T [Tcm], effector memory T [Tem], [Tr1], induced regulatory T cells [iTreg], natural regulatory T cells [nTreg], T helper 1/2/17 cells [Th1/2/17], T follicular helper cells [Tfh], cytotoxic T cells [Tc], mucosal‐associated invariant T cells [MAIT], exhausted T cells [Tex], gamma delta T [γδ T], and NKT cells and six other important immune cells (B cells, macrophages, monocytes, neutrophils, dendritic cells [DC] and NK cells) were estimated using ImmuCellAI (http://bioinfo.life.hust.edu.cn/web/ImmuCellAI/)^[^ ^81^ ^]^

### Dimension Reduction and Clustering Analysis

For SKCM/GSE123139 Seurat, we scaled data with the features calculated by function *FindVariableFeatures*. To remove the batch effects of different samples, we used the *RunHarmony* method in R package *harmony* ^[^ ^82^ ^]^ For clustering and visualization, we applied *FindCluster()* in *Seurat* to obtain cell clusters in various resolutions and reduced the dimensionality of the data using Uniform Manifold Approximation and Projection (UMAP) implemented in *RunUMAP* function with the setting: reduction = “harmony”, dims = 1:10.

### T Cell Classification with ProjectTILs

ProjectTILs was an algorithm that could accurately divide scRNA‐seq data into nine broad T cell subtypes: CD8_NaiveLike, CD8_EarlyActive, CD8_EffectorMemory, CD8_Effector, CD8_Tpex, CD8_Tex, CD4_NaiveLike, Tfh, Th1 and Treg^[^ ^46^ ^]^ After submitting a list of Seurat objects downloaded from TISCH, the ProjectTIL *make.projection* function was applied to normalize scRNA‐seq data by using a log‐transform, automatically removing non‐T cells and projecting the datasets onto a reference map of cellular states. Then, we used the *cellstate.predict* function to annotate each cell. These two functions were conducted with default parameters.

### T Cell Trajectory Analysis Using Monocle and in Line with Hypoxia Score

*Monocle* (version 2.14.0) was used to illustrate the differentiation of CD4^+^ T cells and CD8^+^ T cells in human and mouse tissues^[^ ^83^ ^]^ First, we loaded the normalized count matrices and meta.data information to create a new CellDataSet object. Since the count matrices had been normalized, we used the setting: expressionFamily = uninormal. During construction of the single‐cell trajectories, we first used the *VariableFeatures* function in Seurat (versions 3.2.1) to filter a list of gene ids to be used for defining progress. Then, dimensional reduction was performed using the DDRTree method. Finally, we ordered cells with the state of NaïveLike cells as the root. By means of function *plot_cell_trajectory*, with color_by = “DefineTypes”, “Pseudotime” or “Hypoxia Score”, the result could be visualized. Function *plot_genes_branched_pseudotime* enabled observation of the alteration of hypoxia signatures along with cell trajectory.

### Analysis of T Cell Differentiation‐Related and Hypoxia‐Related TFs

To identify TFs whose differential expression could play a role in T cell development, the *differentialGeneTest* function in monocle was applied to CD4^+^ T and CD8^+^ T Cell Dataset objects, respectively. TFs with p‐value < 0.05 and q‐value < 0.05 were defined as T cell differentiation‐related genes. At the same time, we excluded the TFs whose expression declined along with pseudotime by filtering out those with higher expression at the starting state than at the end state. Moreover, to ensure the consistency of a connection between these TFs and T cell differentiation, we kept TFs that had consistent function in half of all the datasets, which meant 19 CD8^+^ T cell datasets and 17 CD4^+^ T cell datasets. For all the remaining TFs, the Spearman test was used to calculate their correlation with the hypoxia score.

### Analysis of Hypoxia‐Associated Immune Features

The value of aneuploidy, mutation rate, the richness of TCR/BCR, and neoantigen load were obtained from Thorsson et al.^[^ ^84^ ^]^ (https://gdc.cancer.gov/about‐data/publications/panimmune). The immunophenogram score (IPS) was estimated as previously described^[^ ^85^ ^]^ There were four categories that contributed to IPS, including effector cells (activated CD4^+^ T cells [acCD4^+^], activated CD8^+^ T cells [acCD8^+^], effector memory CD4^+^ T cells [Tem CD4^+^], and effector memory CD8^+^ T cells [Tem CD8^+^]), suppressive cells (Tregs and MDSCs), 10 MHC‐related molecules, and 10 checkpoints or immunomodulators. For each determinant, a sample‐based Z score was calculated from the gene expression data. For these six cell types, the average Z‐score from the corresponding metagene was calculated. If the determinant was positive to immune response, the Z‐score was weighted with 1, including each MHC molecule, ICOS, CD27, acCD4^+^, acCD8^+^, Tem CD4^+^, and Tem CD8^+^. If the determinant was negative to immune response, the Z‐score was weighted with −1, including PD‐1, CTLA4, LAG3, TIGIT, TIM3, PD‐L1, PD‐L2, Tregs, and MDSCs. We averaged the weighted Z‐score for the respective category leading to four values, then summed these four averaged weighted Z‐scores, which was considered as IPS.

We used Spearman's correlation to assess the relationship between hypoxia score and immune features, including the abundance of immune cell populations, the silent/non‐silent mutation rate, aneuploidy score, neo‐antigen load, the richness and evenness of TCR/BCR, IPS, and considered false discovery rate (FDR) < 0.05 as significant correlation.

### Evaluation of the Effect of Hypoxia on Immunotherapy

The immunotherapy response was evaluated by RECIST criteria and classified into complete response (CR), partial response (PR), progressive disease (PD) and stable disease (SD)^[^ ^86^ ^]^ For SKCM anti‐PD1 treatment RNA‐seq datasets, we compared the hypoxia score between PD (non‐response group) and PRCR (response group). In IMvigor210 cohort, PRCR and stable disease (SD) were pooled as a response group, while progressive disease (PD) was used for the non‐response group. Statistical analysis was performed by two‐sided Student's *t*‐test. Spearman's correlation was used to perform association analysis of the dynamic alterations of the hypoxia score and immune features for both pre‐ and on‐treatment patient samples.

### Survival Analysis

We used the *maxstat.test* in the R package *maxstat* to divide all samples into hypoxia score‐high and hypoxia score‐low groups, based on the optimal cutpoint. Kaplan–Meier comparative survival analyses for prognostic analysis were generated, and the log‐rank test was used to determine the significance of the differences.

### Cell Lines and Culture Conditions

Raw264.7 cells were obtained from American Type Culture Collection (#TIB‐71, ATCC) and maintained in complete growth medium of ATCC‐formulated Dulbecco's Modified Eagle's Medium (#30‐2002, ATCC) with 10% fetal bovine serum (#A4766801, Gibco) and 1% Penicillin–Streptomycin (#15140122, Gibco). Cells were split into either 6‐well plates or 10 cm dishes and allowed to grow to 70–80% confluence prior to the start of the experiments. For the RNAi experiments, cells were transiently transfected by ON‐TARGETplus Mouse Hif‐1 alpha siRNA Pool (#L‐040638‐00‐0005, Dharmacon) or ON‐TARGETplus Non‐targeting Pool (#D‐001810‐10‐05, Dharmacon) for 48 h with Lipofectamine 3000 (#L3000015, Invitrogen) according to the instructions. For the treatment of HIF‐1 alpha inhibitors, cells were treated with 1 nM of Echinomycin (#ab144247, Abcam) or vehicle for 48 h.

### Induction of Hypoxia

Hypoxia was induced in a CO_2_/O_2_ incubator for hypoxia research (The InvivO2 hypoxia workstation, LAF Technologies). Briefly, cells were cultured in 10 cm dishes at 1% O2 for the time periods specified. Control cells were maintained in normoxic conditions in the same incubator and harvested at the specified times.

### Measurement of mRNA Levels Using Quantitative Real Time PCR (qRT‐PCR)

Total RNA was isolated from Raw264.7 cells in each experimental condition using Quick‐RNA Miniprep Plus Kit (#R1058, Zymo) by following the manufacturer's protocol, and the extracted RNA was quantified using NanoDrop 2000/2000c Spectrophotometers (#ND2000CLAPTOP, Thermo Fisher Scientific, Inc). Total RNA (2–5 µg) was reverse transcribed to cDNA using the iScript Reverse Transcription Supermix for RT‐qPCR (#1708841BUN, Bio‐Rad) according to the manufacturer's recommendation. The cDNA (50–100 ng) was used for real‐time PCR analysis in a final volume of 20 µl containing, iTaq Universal SYBR Green Supermi (#1725125, Bio‐Rad) and specific gene primers for Alcam (Forward: 5′‐ AGGAACATGGCGGCTTCAACGA −3′; Reward: 5′‐ ACACCACAGTCGCGTTCCTACT −3′) or Gapdh (Forward: 5′‐CATCACTGCCACCCAGAAGACTG‐3′; Reward: 5′‐ATGCCAGTGAGCTTCCCGTTCAG‐3′). qPCR was performed using the CFX Connect Real‐time PCR system (Bio‐Rad, United States). Fold changes in expression were calculated using the 2^−ΔΔCt^ method (PMID: 35577506). Each reaction was run in duplicate or triplicate and Gapdh was used as a normalization control.

### Western Blotting

The procedures were performed as described previously^[^ ^87^ ^]^ The primary antibodies used were HIF‐1α (#ab228649, Abcam (1:1000)), CD166 (#ab109215, Abcam (1:1000)), and beta actin (#3700, Cell Signaling Technology (1:1000)). After the washing steps, the membranes were incubated with Goat Anti‐Rabbit IgG (H + L)‐HRP Conjugated‐ (#1706515, Bio‐Rad, 1:10000) or with Goat Anti‐Mouse IgG (H + L)‐HRP Conjugated (#1706516, Bio‐Rad, 1:10000) secondary antibodies and detected using Novex ECL Chemiluminescent Substrate Reagent Kit (#WP20005, Invitrogen).

### Chromatin Immunoprecipitation (ChIP)‐qPCR Assays

We predicted the binding motifs 2 kb upstream and downstream of ALCAM TSS by webtool MEME (https://meme‐suite.org). ChIP assays were performed with HIF1α antibodies according to the EZ‐Magna ChIP A/G Chromatin Immunoprecipitation Kit's protocol (#17‐10086, Sigma). Briefly, 5 × 10^6^ Raw264.7 cells were crosslinked with 1% formaldehyde for 10 min. Cell pellets were incubated in Cell Lysis Buffer for 10 min, and pellet nuclei were harvested by centrifugation at 2000× g and digested in Nuclear Lysis Buffer containing 25 units micrococcal nuclease per IP for 20 min at 37 °C, followed by pulsed ultrasonication to shear cellular DNA and cleared by centrifugation at 12000× g for 10 min. Equal amounts of chromatin were incubated overnight at 4 °C with primary antibody. The following antibodies were adopted: HIF1α (#ab228649, Abcam, 4 µg/IP) and IgG (17‐614, Millipore, 4 µg/IP). DNA pulled down by the antibodies was purified by spin columns, and purified DNA was quantitated by qPCR using CFX Connect Real‐time system (Bio‐Rad). ChIP‐qPCR specific primers for Alcam (Primer#1 Forward: 5′‐TTAGGCTGGCTGCAGTTTGA‐3′; Reward: 5′‐ AGGCTAAATGCTAGGGGCAA‐3′) or (Primer#2 Forward: 5′‐ACTCTCCCTTAGACAAGGTTTCC‐3′; Reward: 5′‐ AACAGTATGTGATTGTGCTGGG‐3′).

### Animal Experiment

All animal experiments were performed in accordance with guidelines approved by MD Anderson's Institutional Animal Care and Use Committee. BALB/c and C57BL/6 were purchased from Jackson Laboratories (Bar Harbor, ME, USA). For establishment of the syngeneic cancer model, 4T1 cells (5×10^4^) and B16F10 cells (5×10^4^) mixed with matrigel were injected into the right inguinal fat pad (4T1) of 6‐week‐old female BALB/cJ mice or into the subcutaneous tissue (B16F10) of 8‐week‐old female C57BL/6 mice. One week after injection, mice were randomly assigned to treatment groups and injected with combinations of the following drugs: scramble or HIF1 LNA (5 mg k^−1^g, SubQ, every other day, QIAGEN) and anti‐PD‐1 (BioXcell, clone RMP1‐14) as ICBs, 100 µg every 72 h, or isotype control antibody (BioXcell, clone LTF‐2) 200 µg every 72 h. Tumors were measured two times per week, and mice were euthanized once the ethical end point was reached. After treatment, mice tumors were collected for immunofluorescence analysis.

### Multiplex Immunohistochemistry

Four µm thick sections of formalin fixed and paraffin embedded (FFPE) tumor tissue on super frost plus slides were de‐paraffinized and rehydrated by serial passage through changes of xylene and graded ethanol. All slides were subjected to a primary heat‐induced epitope retrieval (HIER) in 1 mM EDTA buffer, pH 8.0 at 125 °C for 3 min. Subsequent HIERs were dependent on antibody used and performed in the microwave at 90 °C, 10% power for 15 min. Endogenous peroxidase in tissues was blocked by incubation of slides in 0.3% hydrogen peroxide solution. Antibodies used included rabbit monoclonal CD8 (#98941, Cell Signaling Technology, 1:200), PD‐L1 (#64988, Cell Signaling Technology, 1:200), CD3 (#78588, Cell Signaling Technology, 1:200), CD68 (#26042, Cell Signaling Technology, 1:200), CD4 (#25229, Cell Signaling Technology, 1:200), F4/80(#70076, Cell Signaling Technology, 1:200), EpCAM (#93790, Cell Signaling Technology, 1:200), ALCAM (#ab109215, Abcam, 1:200), HIF1α (#ab228649, Abcam, 1:200), and TIM3 (#83882, Cell Signaling Technology, 1:200). Immunofluorescent signal was visualized using the Opal Polaris 7 Color IHC Detection Kits (#NEL871001KT, Akoyo Biosciences), TSA dyes 480, 520, 570, 620, 690, and 780, counterstained with Spectral DAPI. Color separation, tissue and cell segmentation, and cell phenotyping were performed on inForm Software v2.2 (Perkin Elmer, MA) to extract image data. All slides were scanned at 10× magnification in order to select for high‐powered imaging at 20× (resolution of 0.5 µm per pixel) using Phenochart (Perkin Elmer, MA) or imaged with confocal microscope (Zeiss). Cell segmentation was done based on all cells counter‐stained with DAPI.

### Cell Separation

To separate the cells, the gentleMACS Dissociator from Miltenui Biotec Inc. and the Tumour Dissociation kit from Miltenui Biotec were utilized. This allowed for the isolation of individual cells from the tumor samples. In the case of mouse lymphocytes, the spleen was used as the source for isolation. The EasySep Mouse CD8+ T Cell Isolation Kit was employed to specifically isolate CD8+ T cells. For human PBMCs (peripheral blood mononuclear cells), they were extracted from blood using Lymphoprep. To specifically isolate human CD8+ T lymphocytes, the EasySep Human CD8+ T Cell Isolation Kit was utilized.

### Flow Cytometry

Human and Mouse tumors: human and mouse tumors were dissociated as a single cell using the gentleMACS Dissociator (Miltenui Biotec Inc) with the Tumor Dissociation Kit (Miltenyi Biotec, 130‐095‐929) or mouse Tumor Dissociation kit (Miltenui Biotec), after lysis of red blood cells (RBC Lysis Buffer, BioLegend), single‐cell suspensions were blocked with anti‐CD16/32 (BioLegend) for 10 min on ice and then incubated with appropriate antibodies for 30 min on room temperature. Flow cytometry was performed on an CytoFLEX (Beckman Coulter), and data were analyzed using Cytexpert 2.4. Flow cytometry antibodies were used in the experiments, including CD45 (#304062, Biolegend, diluted 1:100), CD166 (#343906, Biolegend, diluted 1:100; #17‐1661‐82, Invitrogen, diluted 1:100), PD‐1 (#329952, Biolegend, diluted 1:100; #APC‐65142, Proteintech, diluted 1:100), Lag3 (#369306, Biolegend, diluted 1:100; #125208, Biolegend, diluted 1:100), CD68 (#333806, Biolegend, diluted 1:100), and F4/80 (#123130, Biolegend, diluted 1:100).

### Assessment of Proliferation Function

Isolated human and mouse CD8^+^ T were washed and resuspended with 1 ml PBS containing 5% FBS in 15 ml tubes. 1 µl of 5 mM carboxyfluorescein succinimidyl ester (CFSE) solution (Biolegend) was directly added into the cell suspension followed by 5 min incubation at RT in the dark. Centrifuge the cells at 300 g for 5 min to remove the sediment after washing them with ten volumes of 20 °C PBS containing 5% FBS. After staining, 2 × 10^5^ CFSE‐labeled cells were seeded into each well and co‐cultured with *ALCAM* ^high^ and *ALCAM* ^low^ macrophages from human and mouse tumor tissues. Finally, analyze the CFSE‐labeled cells using flow cytometry.

### Luciferase Reporter Assay

The luciferase reporter assay was conducted using the Dual‐Luciferase Reporter Assay System. The cells were co‐transfected with firefly luciferase reporter plasmid (CD166WT, CD166‐Δ1, CD166‐Δ) and renilla luciferase reporter vector pGL‐3 using Lipofectamine2000 from Invitrogen when they reached 70% confluence. Following transfection, the cells were incubated for 24 h. Subsequently, the firefly and renilla luciferase activities were measured using the Dual‐Luciferase Kit from Beyotime Biotechnology (RG027‐1) and the measurements were performed according to the manufacturer's instructions.

## Conflict of Interest

The authors declare no conflict of interest.

## Author Contributions

Z.X., H.Z., and M.S. contributed equally to this work. L.H., Y.Y., Q.H., and C.L. conceived and supervised the project. Z.X., Y.Y., Q.H., and L. H. designed and performed the research. Y.Y., Z.X., and M.S. performed data analysis. H.Z., Q.H., Y.L., C.S., and C.L. developed mouse models and related experiments. L.Y., Y.L., Y.D., Z.J., Q.Z., Y.Y., Q.H., C.L., and L.H. interpreted the results. Y.Y., Z.X., Q.H., Y.L., and L.H. wrote the manuscript with input from all other authors.

## Supporting information

Supporting Information

Supporting Information

## Data Availability

Data sharing is not applicable to this article as no new data were created or analyzed in this study.
