# Supplementary material for: Identification of Hypoxia‐ALCAM high Macrophage‐ Exhausted T Cell Axis in Tumor Microenvironment Remodeling for Immunotherapy Resistance
Source: Adv Sci (Weinh). 2024 Jul 2;11(33):2309885. doi: 10.1002/advs.202309885 (PMC11434037; doi:10.1002/advs.202309885)
Supplement: Supplementary file 1 — Supporting Information [file ADVS-11-2309885-s002.pdf]

ADVANCED SCIENCE
Open Access

Supporting Information

for *Adv. Sci.*, DOI 10.1002/advs.202309885

Identification of Hypoxia-*ALCAM*^{high} Macrophage- Exhausted T Cell Axis in Tumor Microenvironment Remodeling for Immunotherapy Resistance

Zhenzhen Xun, Huanran Zhou, Mingyi Shen, Yao Liu, Chengcao Sun, Yanhua Du, Zhou Jiang, Liuqing Yang, Qing Zhang, Chunru Lin, Qingsong Hu*, Youqiong Ye* and Leng Han**

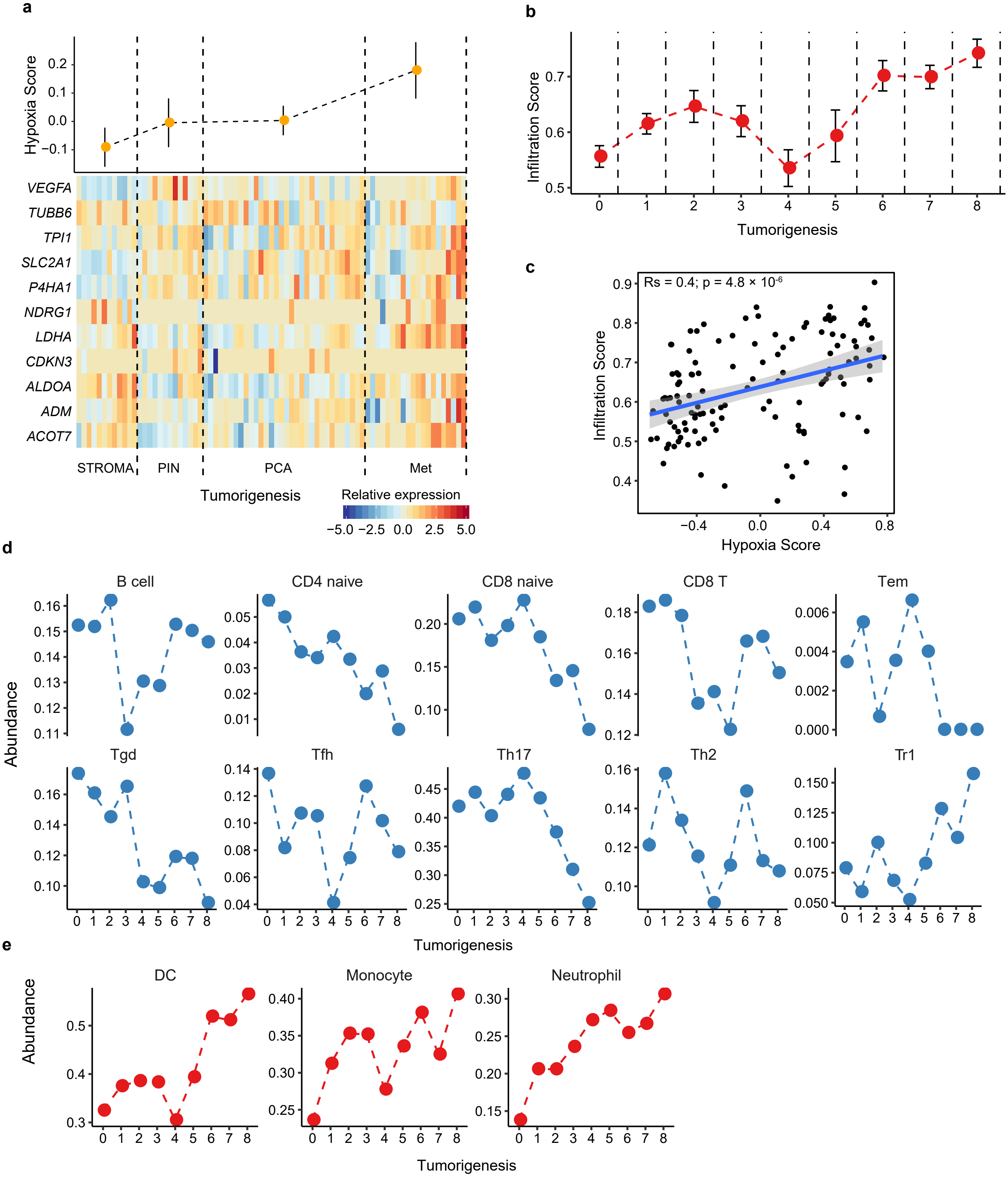

(Legend on next page)

Supplementary Figure 1. Alteration of hypoxia score and immune infiltration during tumor developmental stages

(a) Continuous shift of hypoxia score (upper panel) during prostate carcinogenesis. Heatmaps (bottom panel) show the expression level of 15 genes of hypoxia signature for each stage.
(b) Continuous shift of immune infiltration score during lung carcinogenesis calculated by ImmuCellAI.
(c) The Spearman's correlation of hypoxia score and infiltration score in LSCC.
(d-e) The abundance of immune cells negatively **(d)** or positively **(e)** correlated to hypoxia score in each developmental stage.
Abbreviation: STROMA, stromal nodules of benign prostatic hyperplasia; PIN, prostatic intraepithelial neoplasia; PCA, prostate carcinoma; MET, metastatic prostate cancer; CD8 naive, CD8 naive T cells; CD4 naïve, CD4 naïve T cells; Tgd, Gamma delta T cells; CD8 T, CD8 T cells; Tem, effector memory T cells; Th2/Th17: T helper 2/17 cells; Tfh, T follicular helper cells; DC, dendritic cells.

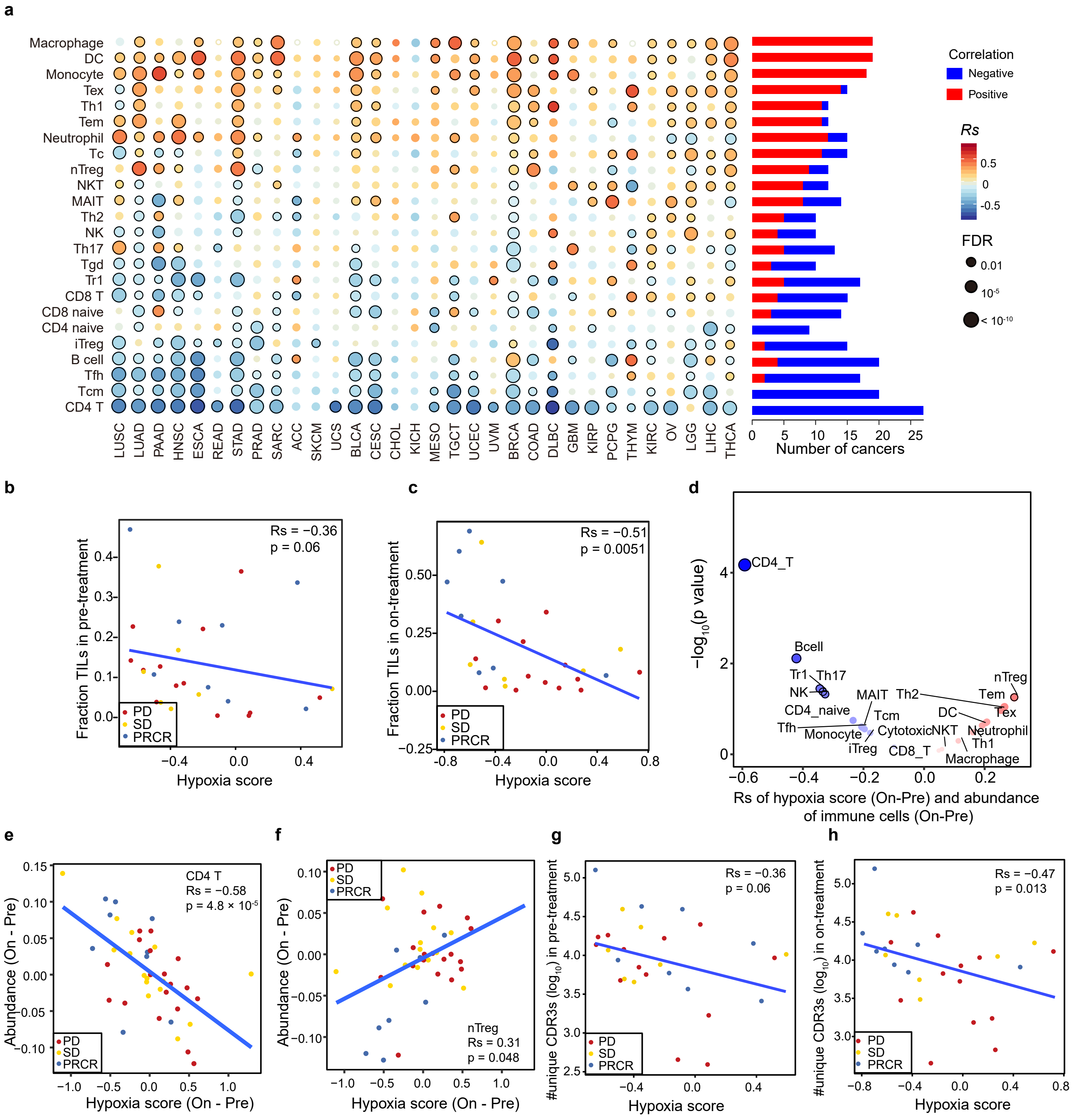

(Legend on next page)

Supplementary Figure 2. Correlation of hypoxia with immune features and treatment response.
(a) The Spearman's rank correlation of hypoxia score and the abundance of immune cell populations across 32 cancer types. The bar plots in the right panel indicate the number of cancer types with positive and negative correlation between hypoxia score and the abundance of immune cells. The cancer types (x-axis) are ordered by the number of immune cells with positive correlation minus the number with negative correlation. (y-axis) are ordered by the number of cancer types with positive correlation minus the number with negative correlation. Abbreviation: CD8 naive, CD8 naive T cells; CD4 naive, CD4 naive T cells; Tgd, Gamma delta T cells; CD8 T, CD8 T cells; Tem, effector memory T cells; Th2/Th17: T helper 2/17 cells; Tfh, T follicular helper cells; DC, dendritic cells; iTreg, induced regulatory T cells.
(b-c) The Spearman's correlation of tumor-infiltrating lymphocytes (TILs) and hypoxia score in pre-treatment **(b)** and on-treatment **(c)** samples. Blue, PR/CR (partial or complete response); Red, PD (progressive disease); Yellow, SD (stable disease).
(d) The Spearman's correlation of alteration of hypoxia score and the alternation of each immune cell abundance.
(e-f) The Spearman's correlation of alteration of hypoxia score and the abundance alteration of CD4 T cells **(e)** and nTregs **(f)** between on-treatment and pre-treatment samples.
(g-h) The Spearman's correlation of CDR3s and hypoxia score in pre-treatment **(g)** and on-treatment **(h)**.

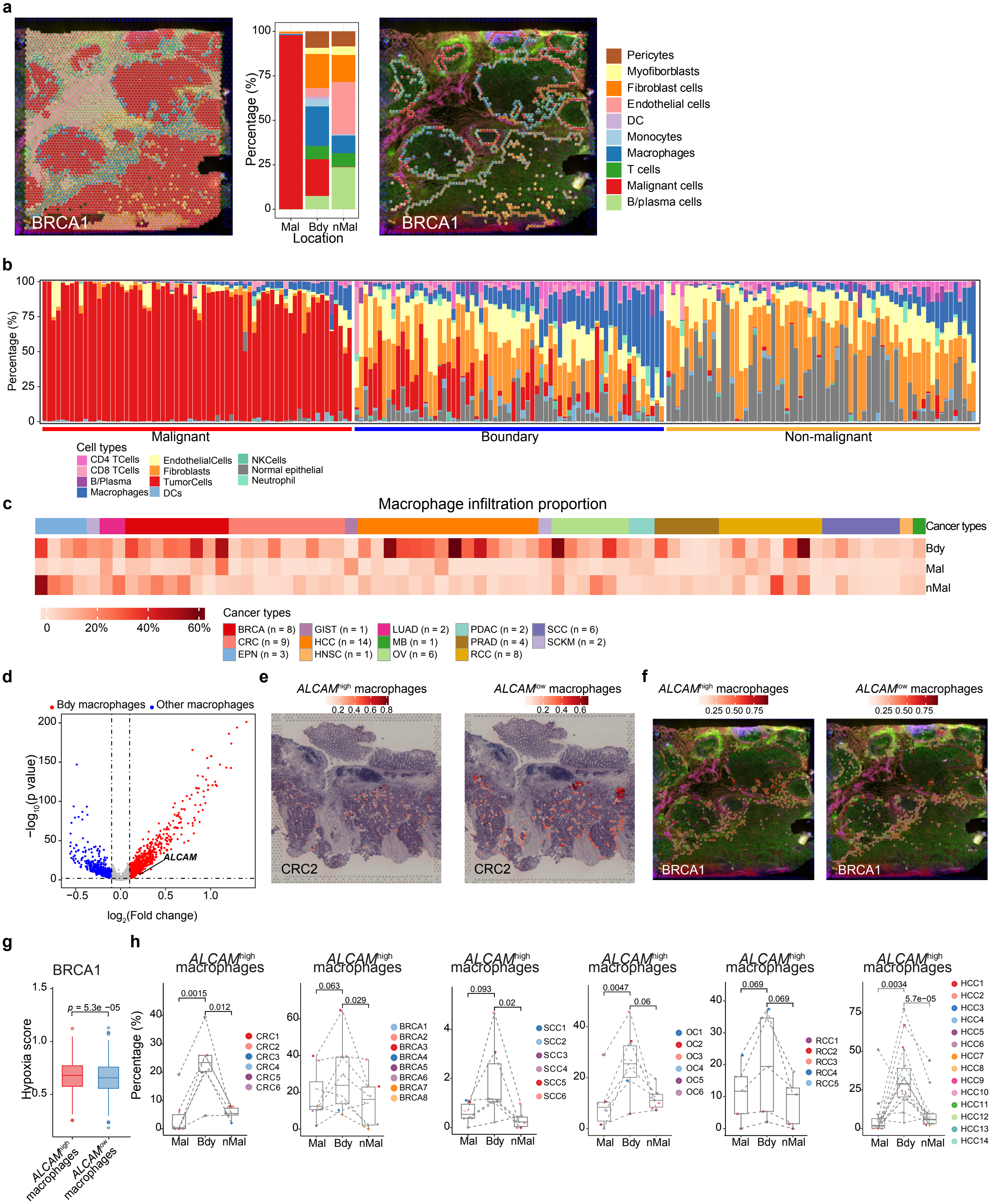

a
BRCA1
Percentage (%)
Mal
Bdy
nMal
Location
Pericytes
Myofibroblasts
Fibroblast cells
Endothelial cells
DC
Monocytes
Macrophages
T cells
Malignant cells
B/plasma cells
b
Percentage (%)
Malignant
Boundary
Non-malignant
Cell types
CD4 TCells
CD8 TCells
B/Plasma
Macrophages
EndothelialCells
Fibroblasts
TumorCells
DCs
NKCells
Normal epithelial
Neutrophil
c
Macrophage infiltration proportion
Cancer types
Bdy
Mal
nMal
0
20%
40%
60%
BRCA (n = 8)
CRC (n = 9)
EPN (n = 3)
GIST (n = 1)
HCC (n = 14)
HNSC (n = 1)
LUAD (n = 2)
MB (n = 1)
OV (n = 6)
PDAC (n = 2)
PRAD (n = 4)
RCC (n = 8)
SCC (n = 6)
SCKM (n = 2)
d
Bdy macrophages
Other macrophages
−log10(p value)
ALCAM
log2(Fold change)
e
ALCAMhigh macrophages
ALCAMlow macrophages
CRC2
f
BRCA1
g
BRCA1
Hypoxia score
p = 5.3e −05
ALCAMhigh macrophages
ALCAMlow macrophages
h
ALCAMhigh macrophages
Percentage (%)
0.0015
0.012
0.063
0.029
0.093
0.02
0.0047
0.06
0.069
0.069
0.0034
5.7e−05
CRC1
CRC2
CRC3
CRC4
CRC5
CRC6
BRCA1
BRCA2
BRCA3
BRCA4
BRCA5
BRCA6
BRCA7
BRCA8
SCC1
SCC2
SCC3
SCC4
SCC5
SCC6
OC1
OC2
OC3
OC4
OC5
OC6
RCC1
RCC2
RCC3
RCC4
RCC5
HCC1
HCC2
HCC3
HCC4
HCC5
HCC6
HCC7
HCC8
HCC9
HCC10
HCC11
HCC12
HCC13
HCC14

Supplementary Figure 3. Spatial location of *ALCAM*^{high} macrophages

(a) Spatial scatter pie plots representing the proportions of the seven cell types predicted by Cottrazm in whole BRCA ST slide spots (left). Bar plots representing the proportions of the seven cell types predicted by Cottrazm in spots from malignant, tumor boundary, and non-malignant regions (middle). Spatial scatter pie plots representing the proportions of the seven cell types predicted by Cottrazm in tumor boundary spots (right).

(b) Bar plot showing the cellular composition of malignant region, tumor boundary, and non-malignant region in 68 tumor samples across 14 cancer types.

(c) Heatmap showing the infiltration of macrophage in each spatial region in each sample.

(d) Volcano plot exhibiting the differentially expressed genes from macrophage tumor boundary and other remained regions in BRCA ST slides.

(e-f) Predicted proportion within each capture spot for *ALCAM*^{high} macrophages (left) and *ALCAM*^{low} macrophages (right) in CRC **(e)** and BRCA **(f)**.

(g) Boxplots showing the hypoxia score among *ALCAM*^{high} macrophages and *ALCAM*^{low} macrophages in BRCA ST slide.

(h) Box plots showing the mean proportion of ALCAMhigh macrophages in each spatial region in six cancer types. A paired t-test was performed to assess the statistical significance. The boxes in **g, h** showed the median ±1 quartile, with the whiskers extending from the hinge to the smallest or largest value within 1.5× the IQR from the box boundaries. A two-sided Wilcoxon signed-rank test was used to assess statistical significance.

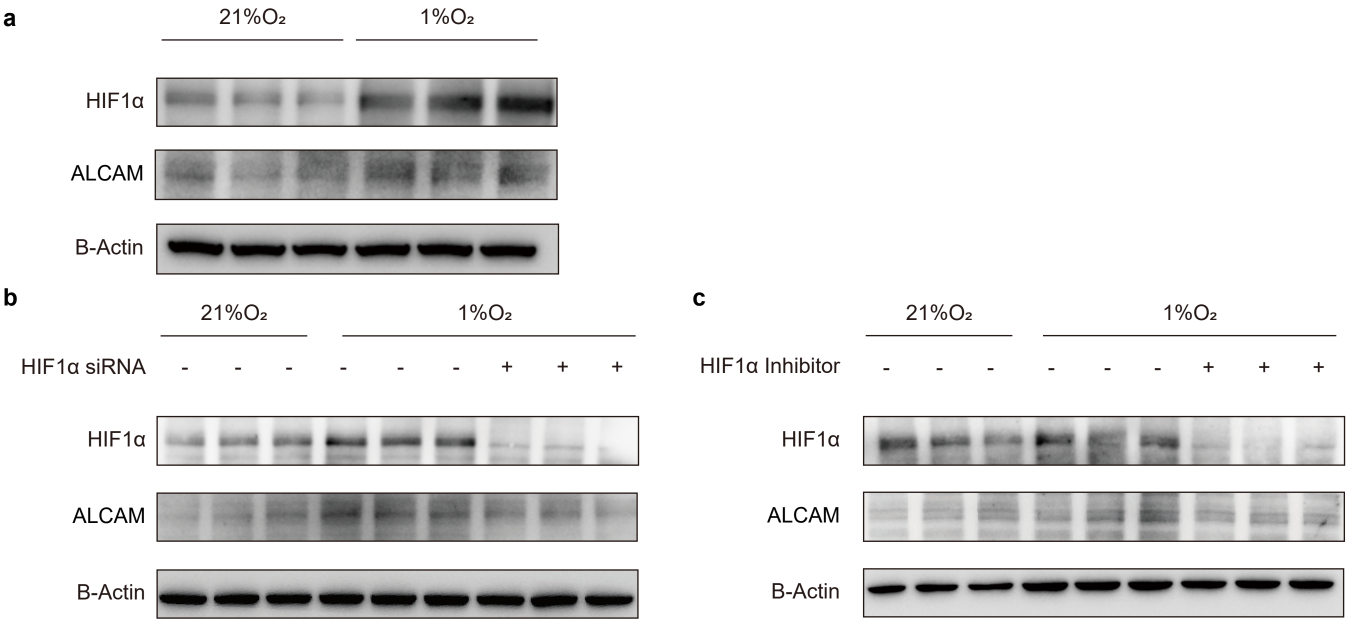

Supplementary Figure 4. HIF1α regulates ALCAM expression on macrophages.
(a) Western blot showing the protein level of Hif1a and Alcam in Raw264.7 cells with 21% O_2 and 1% O_2. **(b-c)** Western blot showing the protein level of Hif1a and Alcam in Raw264.7 cells with 21% O_2, 1% O_2 and 1% O_2 under with Hif1a siRNA **(b)** and inhibitor **(c)**.

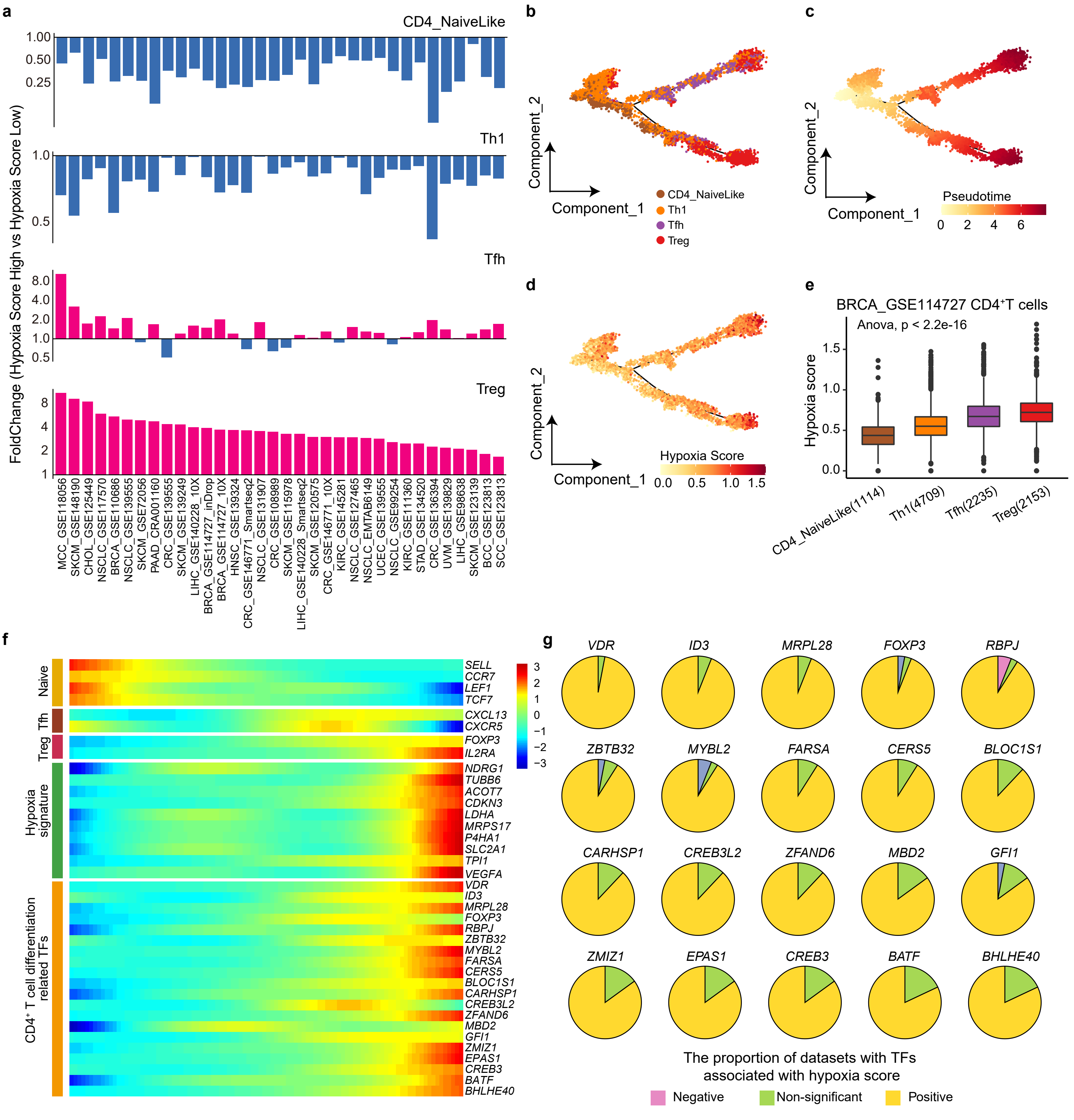

(Legend on next page)

Supplementary Figure 5. Association analysis of hypoxia score and $CD4^+$ T cell differentiation in human cancers

(a) Bar plot showing the fold change for $CD4^+$ T cell subset proportion when hypoxia status changes.
(b) Differentiation trajectory of $CD4^+$ T cell subsets inferred by Monocle2.
(c) The pseudotime of BRCA $CD4^+$ T cells.
(d) Dynamic alteration of hypoxia score during BRCA $CD4^+$ T cell differentiation.
(e) Box plot with hypoxia score for $CD4^+$ T cell subsets in an increasing order during the differentiation.
(f) Box plot with hypoxia score for $CD4^+$ T cell subsets in an increasing order.
(g) Heatmap of gene differential expression along with $CD4^+$ T cell differentiation.
The boxes in **e** showed the median ±1 quartile, with the whiskers extending from the hinge to the smallest or largest value within 1.5× the IQR from the box boundaries. A one-way ANOVA test was performed in **e**.

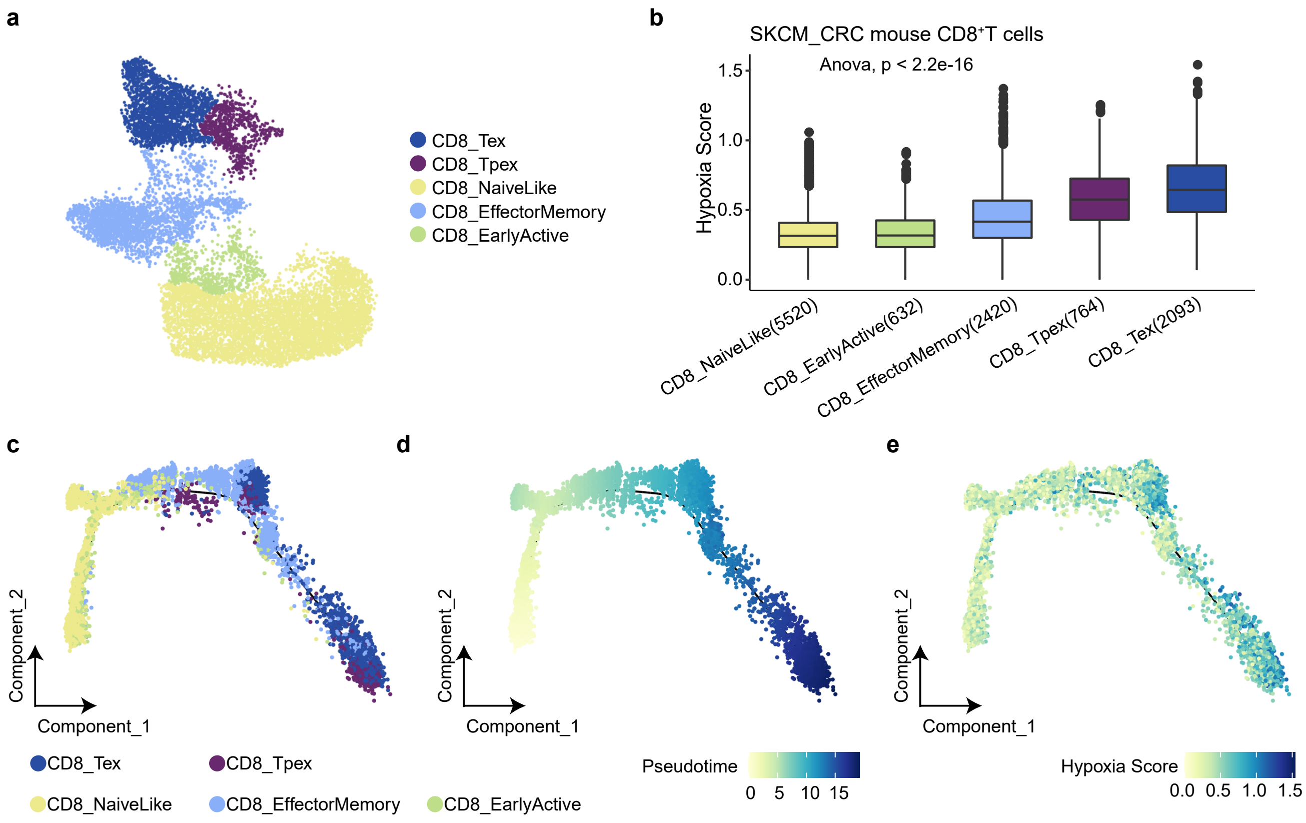

Supplementary Fig. 6. Association analysis of hypoxia score and CD8⁺ T cell differentiation in melanoma and colon cancer mouse model

(a) UMAP plot showing clusters of CD8⁺ T cells for melanoma and colon cancer mouse model.
(b) Box plot with hypoxia score for CD8⁺ T cell subsets in an increasing order.
(c) Developmental trajectory of CD8⁺ T cell subsets inferred by Monocle.
(d) Mouse CD8⁺ T cells which are ordered by pseudotime.
(e) Dynamic alteration of hypoxia score during mouse CD8⁺ T cell differentiation.
The boxes in **b** showed the median ±1 quartile, with the whiskers extending from the hinge to the smallest or largest value within 1.5× the IQR from the box boundaries. A one-way ANOVA test was performed in **b**.

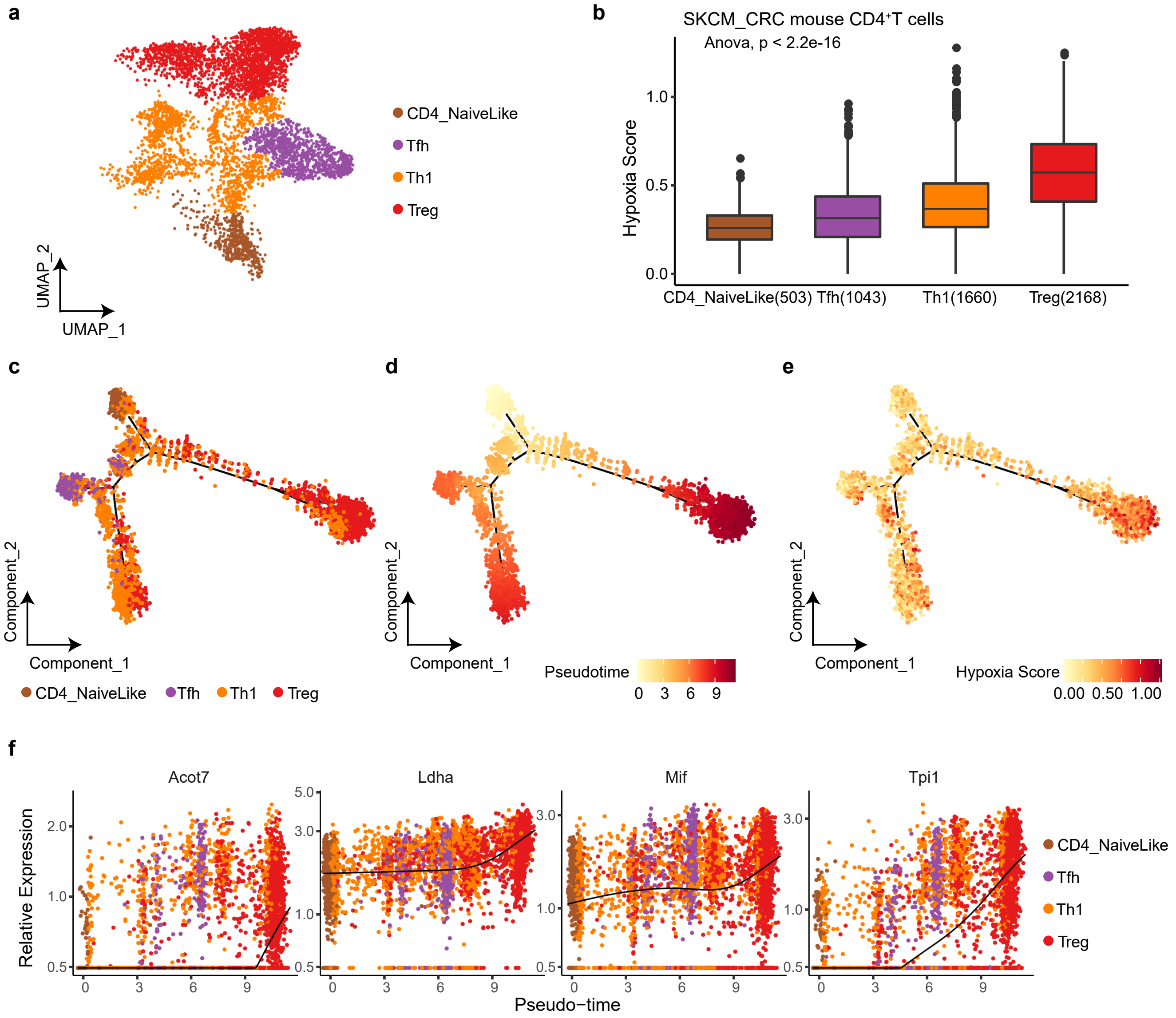

Supplementary Fig. 7. Association analysis of hypoxia score and CD4⁺ T cell differentiation in melanoma and colon cancer mouse model

(a) UMAP plot showing clusters of CD4⁺ T cells for melanoma and colon cancer mouse model.
(b) Box plot with hypoxia score for CD4⁺ T cell subsets in an increasing order.
(c) Developmental trajectory of CD4+ T cell subsets inferred by Monocle.
(d) Mouse CD4⁺ T cells which are ordered by pseudotime.
(e) Dynamic alteration of hypoxia score during h mouse CD4⁺ T cell differentiation.
(f) Change in expression levels of hypoxia signatures (Aldoart1, Eno1b, Pgam1 and Tpi1) through the pseudotime order of CD4⁺ T cells.
The boxes in **b** showed the median ±1 quartile, with the whiskers extending from the hinge to the smallest or largest value within 1.5× the IQR from the box boundaries. A one-way ANOVA test was performed in **b**.

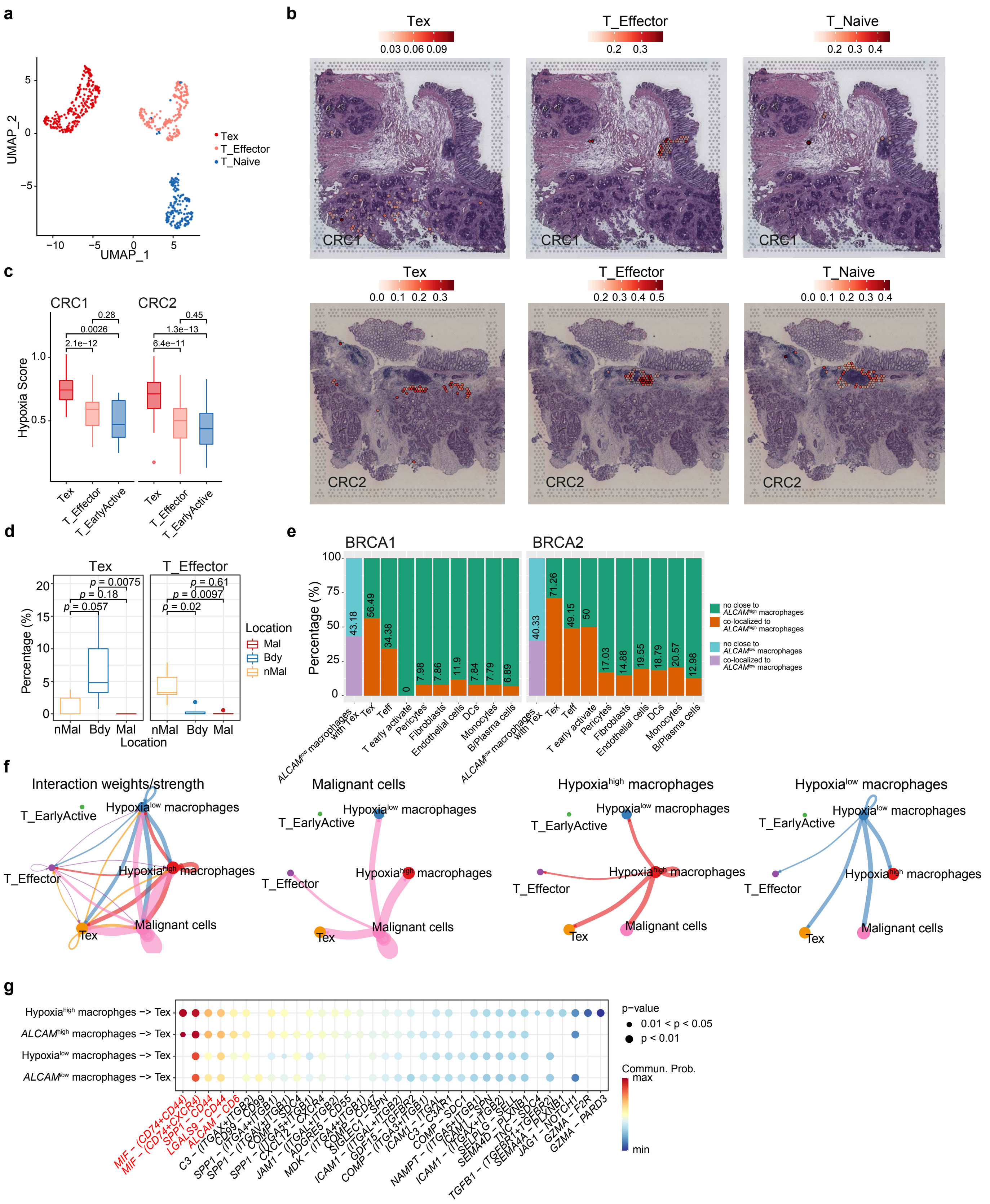

(Legend on next page)

Supplementary Fig. 8. The spatial location of T cell subtypes in CRC

(a) The characteristics of T cell subtypes in the ST dataset. The UMAP projections of subtypes of myeloid cells in CRC, including T-PRF1, T-IER3, and Tex (exhausted CD8 T cells).

(b) Predicted proportion within each capture spot for T-PRF1, T-IER3, and Tex in CRC.

(c) The exhaustion score among T-PRF1, T-IER3, and Tex in two CRC ST slides.

(d) Box plots showing proportion of macrophage subtypes in each region. The boxes in d showed the median ±1 quartile, with the whiskers extending from the hinge to the smallest or largest value within 1.5× the IQR from the box boundaries. A one-way ANOVA test was performed in d.

(e) Bar plots showed the co-localization percentage among *ALCAM*$^{high/low}$ macrophages with Tex and other cell types in BRCA1 (left) and BRCA2 (right).

(f) Circle plot representing the cellular communication of cell types among malignant cells, Hypoxia^{high} macrophages, Hypoxia^{low} macrophages, T_EarlyActive, T_Effector, and Tex. Circle sizes are proportional to the number of cells in each cell group and thickness of the flow represents the interaction weight.

(g) All the significant ligand-receptor pairs that contribute to the signaling sending from *ALCAM*^{high} macrophages and Hypoxia^{high} macrophages to exhausted T cells. to three T cell subtypes. The dot color and size represent the calculated communication probability and p-values. p-values are computed from one-sided permutation test.

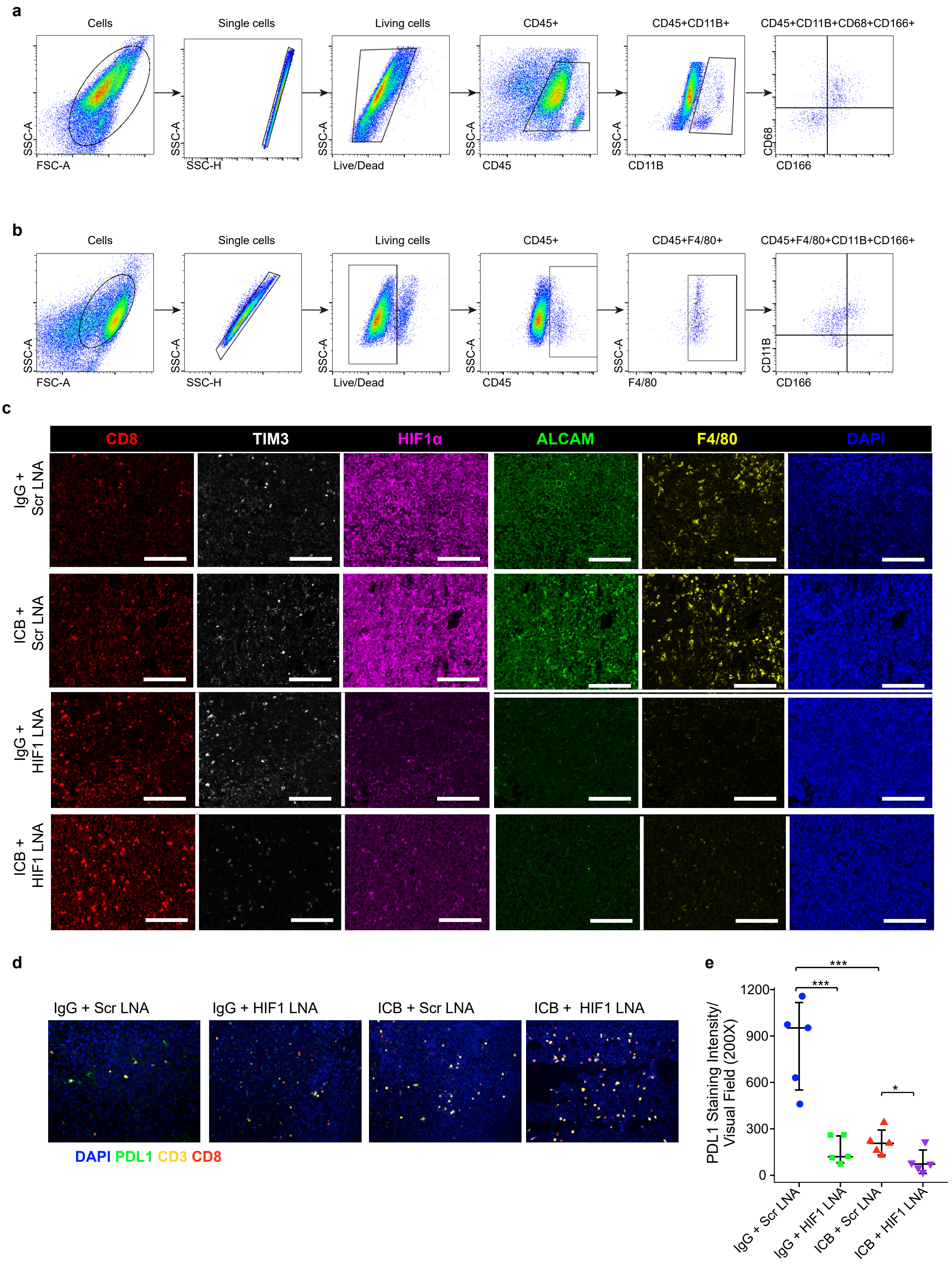
a
Cells
Single cells
Living cells
CD45+
CD45+CD11B+
CD45+CD11B+CD68+CD166+
SSC-A
FSC-A
SSC-H
Live/Dead
CD45
CD11B
CD68
CD166
b
Cells
Single cells
Living cells
CD45+
CD45+F4/80+
CD45+F4/80+CD11B+CD166+
SSC-A
FSC-A
SSC-H
Live/Dead
CD45
F4/80
CD11B
CD166
c
CD8
TIM3
HIF1α
ALCAM
F4/80
DAPI
IgG + Scr LNA
ICB + Scr LNA
IgG + HIF1 LNA
ICB + HIF1 LNA
d
IgG + Scr LNA
IgG + HIF1 LNA
ICB + Scr LNA
ICB + HIF1 LNA
DAPI PDL1 CD3 CD8
e
PDL1 Staining Intensity/ Visual Field (200X)
1200
900
600
300
0
***
***
*
IgG + Scr LNA
IgG + HIF1 LNA
ICB + Scr LNA
ICB + HIF1 LNA

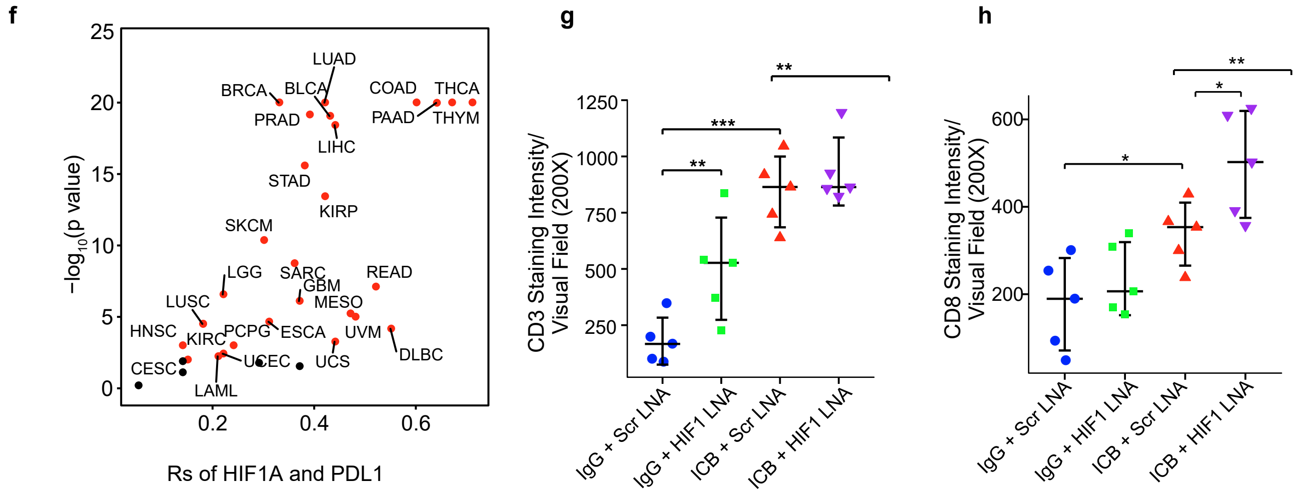

Supplementary Figure 9. The immune features for anti-HIF1A combined with PD-1 blockade.
(**a-b**) Gating strategy.
(**c-d**) Representative images CD8, TIM3, ALCAM (**c**) and PD-L1 (**d**) of syngeneic 4T1 mice model treated scramble or HIF1 LNA, ICB alone or in combination.
(**e**) Statistical analysis of PD-L1 of syngeneic 4T1 mice model treated scramble or HIF1 LNA, ICB alone or in combination (n=7, 7, 7, 7 animals).
(**f**) The Spearman's correlation of mRNA expression between HIF1A and PD-L1 across 33 cancer types.
(**g-h**) Statistical analysis of CD3 (**g**) and CD8 (**h**) of syngeneic 4T1 mice model treated scramble or HIF1 LNA, ICB alone or in combination (n=7, 7, 7, 7 animals). (**c-d**) Scale bars; 100μm. (**e**, **g-h**) (**p<0.01, ***p<0.001). Results are mean ± s.d. of n = 3 independent experiments yielding similar results, P values were determined by one-way ANOVA.
